# Targeting the DNA Damage Response and DNA Repair Pathways to Enhance Radiosensitivity in Colorectal Cancer

**DOI:** 10.3390/cancers14194874

**Published:** 2022-10-05

**Authors:** Siyao Deng, Tijana Vlatkovic, Moying Li, Tianzuo Zhan, Marlon R. Veldwijk, Carsten Herskind

**Affiliations:** 1 Cellular and Molecular Radiation Oncology Lab, Department of Radiation Oncology, Universitätsmedizin Mannheim, Medical Faculty Mannheim, Heidelberg University, Theodor-Kutzer-Ufer 1-3, 68167 Mannheim, Germany; 2 Department of Medicine II, Universitätsmedizin Mannheim, Medical Faculty Mannheim, Heidelberg University, 68167 Mannheim, Germany

**Keywords:** colorectal cancer, radiosensitivity, DNA damage response, DNA repair, cell-cycle checkpoint inhibitors

## Abstract

**Simple Summary:**

The DNA damage response pathway plays a critical role in maintaining genomic integrity. Therefore, inhibition of activation of cell-cycle checkpoints involved in this pathway may increase the sensitivity of tumor cells to DNA damage induced by ionizing radiation. In this review, we provide an overview of mechanisms, preclinical studies and advances in clinical trials of DNA-PKcs, ATM/ATR, CHK1/CHK2, WEE1 and PARP1 kinase inhibitors combined with radiotherapy for colorectal cancer treatment. We evaluate the potential of developing high-efficiency and low-toxicity radiosensitizers targeting the DNA damage response and DNA repair pathways to enhance the response to radiotherapy in colorectal cancer.

**Abstract:**

Radiotherapy is an important component of current treatment options for colorectal cancer (CRC). It is either applied as neoadjuvant radiotherapy to improve local disease control in rectal cancers or for the treatment of localized metastatic lesions of CRC. DNA double-strand breaks (DSBs) are the major critical lesions contributing to ionizing radiation (IR)-induced cell death. However, CRC stem cells promote radioresistance and tumor cell survival through activating cell-cycle checkpoints to trigger the DNA damage response (DDR) and DNA repair after exposure to IR. A promising strategy to overcome radioresistance is to target the DDR and DNA repair pathways with drugs that inhibit activated cell-cycle checkpoint proteins, thereby improving the sensitivity of CRC cells to radiotherapy. In this review, we focus on the preclinical studies and advances in clinical trials of DNA-dependent protein kinase catalytic subunit (DNA-PKcs), ataxia telangiectasia mutated (ATM), ataxia telangiectasia and Rad3-related kinase (ATR), checkpoint kinase 1 (CHK1), checkpoint kinase 2 (CHK2), WEE1 and poly (ADP-ribose) polymerase 1 (PARP1) kinase inhibitors in CRC. Importantly, we also discuss the selective radiosensitization of CRC cells provided by synthetic lethality of these inhibitors and the potential for widening the therapeutic window by targeting the DDR and DNA repair pathways in combination with radiotherapy and immunotherapy.

## 1. Introduction

Colorectal cancer (CRC) is the third most frequent cancer globally and the second major cause of cancer-related fatalities, accounting for roughly 900,000 deaths each year, with the incidence expected to rise to 2.5 million new cases in 2035 [1,2]. Depending on the stage, location and lymph node status of the disease, established treatments for CRC are surgery, chemotherapy, with or without concomitant radiotherapy, and targeted therapies. The 5-year survival rate is >90% for patients with localized CRC but declines sharply to 11–15% for patients suffering from metastatic CRC (mCRC) [3]. Currently, the most common treatment for mCRC patients is a combination chemotherapy containing 5-fluorouracil (5-FU), oxaliplatin and/or irinotecan. In addition, depending on the genotype of the tumor and its location, antibodies targeting the vascular endothelial growth factor (such as bevacizumab) or epidermal growth factor receptor (such as cetuximab) are added to the chemotherapy regimen [4,5]. However, treatment resistance is a major therapeutic challenge. Currently, for patients with locally advanced rectal cancer, the standard therapy is neoadjuvant chemoradiotherapy followed by surgery [6,7]. Although combined chemoradiotherapy improves the pathological complete response (pCR) and local control compared to radiotherapy alone, it also increases toxicity in the normal tissue, as conventional chemotherapy does not specifically target tumor cells [6,7].

Radiotherapy induces DNA damage, including base damage, single-strand breaks (SSBs), double-strand breaks (DSBs) and inter-strand cross-links (ICLs), with unrepaired or misrepaired DSBs being the major lesions responsible for ionizing radiation (IR)-induced cell death. Cancer stem cells (CSCs) are considered an important factor in tumor radioresistance, contributing to failure of radiotherapy [8,9,10,11]. Differentiated non-CSCs tumor cells commonly undergo apoptosis after radiation, while colorectal CSCs may evade radiation-induced cell death by a variety of mechanisms, including altering the DNA damage response (DDR) and DNA repair pathways [12,13]. For example, radioresistant CSCs derived from colorectal HCT116 cells showed enhanced survival associated with a significantly reduced number of phosphorylated histone γH2AX repair foci and increased mRNA and protein expression levels of ERCC1, a subunit of the repair endonuclease, XPF-ERRC1 [14].

Here we provide an overview of the IR-induced DDR and DNA repair pathways and assess the potential of developing high-efficiency and low-toxicity radiosensitizers targeting the DDR and DNA repair pathways in preclinical studies and clinical trials to enhance the radiosensitivity in CRC.

## 2. The DNA Damage Response (DDR)

The DDR is a complex network of cell-cycle arrest, DNA repair and clonogenic inactivation with several interconnected signaling pathways and mechanisms aimed at maintaining cell viability and avoiding tumorigenesis. These include DNA damage recognition, cell-cycle checkpoint activation and arrest, DNA repair, chromatin remodeling, metabolism, and apoptosis [15]. Identifying and repairing damaged DNA necessitates the activation of a carefully regulated set of activities. Thus, DNA damage detection activates cell-cycle checkpoints, which halt the cell cycle for DNA repair prior to cell division allowing cells to survive genome instability and replication stress, and guiding irreparably damaged cells towards permanent arrest or programmed death.

The DDR signaling pathway is made up of a series of reactions with distinct sets of proteins specialized for certain types of damage, which may be classified as sensors, transducers and effectors [16]. DSBs are quickly sensed by the Mre11–Rad50–Nbs1 (MRN) complex, which then interacts with chromatin, followed by promoting the activation of ataxia telangiectasia mutated (ATM) kinase through rapid autophosphorylation at the Ser1981 site [17]. ATM triggers signal transduction by activating the phosphorylation of hundreds of substrates, including the transcription factor p53 (TP53) and the checkpoint kinase 2 (CHK2), inducing cell-cycle arrest and apoptosis [18]. Additionally, ATM enables the phosphorylation of histone H2AX to produce γH2AX as well, which is essential for the coordination of cell-cycle checkpoint activation and DSBs repair [19]. Unlike DSBs, SSBs are detected by the Rad9–Hus1–Rad1 complex, which activates ataxia telangiectasia and Rad3-related kinase (ATR) in collaboration with Rad17, Rfc2, Rfc3, Rfc4 and Rfc5 [20]. ATR is directed to replication protein A (RPA)-encapsulated single-strand DNA through its subunit ATR-interacting protein. After this induction step, Rad9 combines with its partner protein DNA topoisomerase 2-binding protein 1 (TopBP1), which leads to ATR-mediated phosphorylation of the checkpoint kinase 1 (CHK1). CHK1 and CHK2 mediate signals from sensors and phosphorylate the various effectors downstream. CHK2 suppresses CDC25A, a phosphatase that eliminates the suppressive phosphorylation of cyclin E/cyclin-dependent kinase (CDK) 2 and cyclin A/CDK2 complexes, thereby blocking cells in the G1 phase from entering the S phase [21]. CHK1 regulates the G2/M checkpoint by activating WEE1 kinase, which then phosphorylates CDK1, decreasing its activity and inhibiting entrance to mitosis. Moreover, CHK1 modulates the S-phase checkpoint through facilitating the degradation of CDC25A phosphatase, the activity of which is critical for the removal of suppressive phosphate groups of CDK4 and CDK2 kinases and ensuring cell-cycle progression [22].

**Figure 1:**
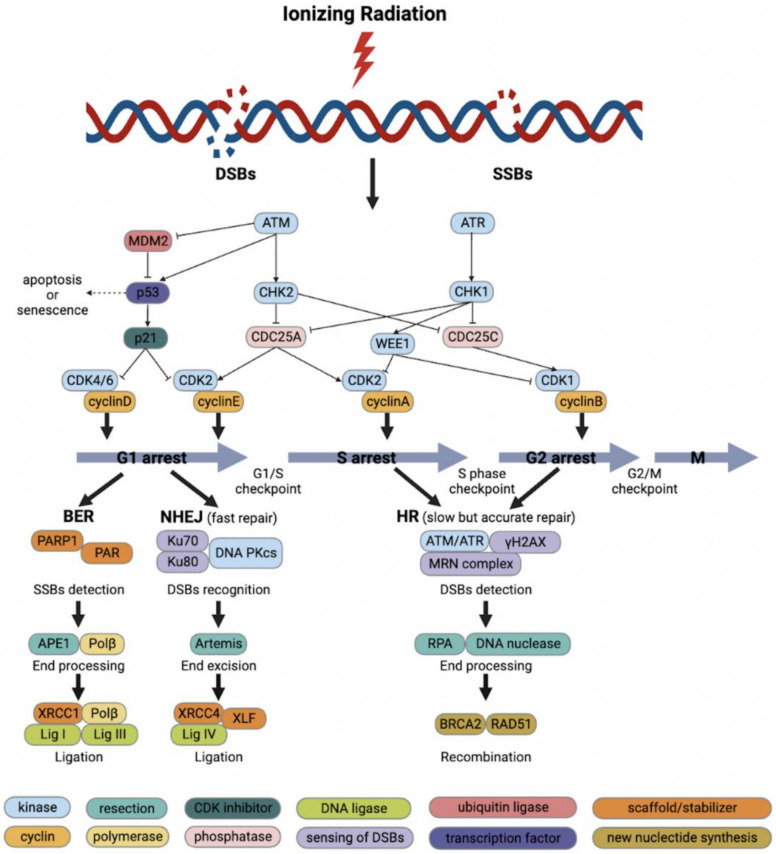
Schematic diagram of ionizing radiation (IR)-induced DNA damage response and DNA repair. Cell-cycle checkpoints are activated in response to IR-induced DNA damage. ATM kinase is activated primarily by DNA double-strand breaks (DSBs) and mediates the initial response to DSBs as well as cell-cycle arrest through activation of CHK2. P53 activates the G1/S checkpoint via p21 to promote DNA repair or to induce apoptosis or senescence. DNA single-strand breaks (SSBs) activate ATR kinase, which in turn activates the S-phase checkpoint and G2/M checkpoint through the action of CHK1 and WEE1. The base excision repair (BER) repairs SSBs and base damage with fast kinetics. Non-homologous end-joining (NHEJ) repairs most direct 2-ended IR-induced DSBs and is the main pathway to repair DSBs in the G1 phase. Homologous recombination (HR) is essential for the repair of complicated DSBs and can only function in the presence of sister chromatids during the S and G2 phase.

Cell-cycle checkpoints can be activated during the G1- to S-phase transition, S phase and G2- to M-phase transition as a response to DNA damage (Figure 1) [23]. Since cell-cycle progression is controlled by CDKs and their endogenous inhibitors, the DDR pathway eventually converges on the regulation of CDK activity. During the G1/S phase, cyclin D binds CDK4 or CDK6 to produce cyclin D/CDK4 or cyclin D/CDK6 complex that induces phosphorylation of retinoblastoma protein (pRB), resulting in the release of transcription factor E2F from pRB and triggering the transcription of cyclin E. Subsequently, cyclin E further binds CDK2 to produce cyclin E/CDK2 complex, which continues to phosphorylate pRB and increase the activity of the S phase in a positive feedback loop to facilitate the G1 to S transition [24,25]. Notably, the G1 checkpoint is heavily dependent on p53, which plays a crucial function in protecting hereditary stability by prevention of mutations and mediation of tumor inhibition through a strictly controlled network [26]. Under physiological circumstances, p53 protein is maintained at low level by tight binding to the murine double minute 2 (MDM2), a p53-specific E3 ubiquitin ligase, resulting in its proteasomal degradation [27]. The G1/S checkpoint is activated via the ATM/p53/p21 pathway by DNA damage in the G1 phase [28]. First, BRCA1 is phosphorylated by ATM at the Ser1423 and Ser1524 sites, and subsequent activation of ATM by BRCA1 phosphorylation causes p53 to be phosphorylated at the Ser15 site, decreasing its binding to MDM2, thus stabilizing p53 expression at the post-transcriptional level [29,30]. Stable p53 stimulates transcription of the downstream gene *CDKN1A* coding for the CDK inhibitor p21, which binds and further represses the cyclin E/CDK2 and cyclin A/CDK2 complexes, leading to the G1/S arrest [31,32]. Tumor cells are usually defective in the G1-phase checkpoint because of the high frequency of *TP53* mutations, especially in CRC with frequencies up to 60% [33]. As a result, *TP53*-deficient cells rely on the S- and G2/M-phase checkpoints activated by ATM and ATR for DNA repair.

When cells undergo DNA damage in the S phase, stalled replication forks, DSBs, SSBs and ICLs can all trigger temporary S-phase arrest to block further replication [34,35]. ATR and ATM are activated by DNA damage and promote the proteasomal degradation of CDC25A via CHK1 and CHK2, respectively, thereby suppressing the activity of cyclin A/CDK2 complex and preventing the further progression of the S phase [21,36]. In addition, ATR and ATM trigger the G2/M checkpoint, thus preventing cells with DNA damage from entering into mitosis. If DSBs are detected, ATM phosphorylates CHK2, thereby inhibiting CDC25C phosphatase activity, which contributes to the phosphorylation and inactivation of CDK1. Moreover, ATR contributes to delayed arrest in the G2 phase. Upon recognition of SSBs, ATR phosphorylates CHK1, thereby activating WEE1 and inhibiting CDC25C activity, which further prevents the activation of cyclin B/CDK1 complex and results in the G2-phase arrest [37]. WEE1 and protein kinase membrane-associated tyrosine/threonine 1 (PKMYT1) negatively regulate the G2 to M transition by functioning in the cyclin B/CDK1 complex [38]. WEE1 phosphorylates CDK1 at Tyr15 of its catalytic subunit, whereas PKMYT1 phosphorylates CDK1 both at the residues Tyr15 and Thr14 resulting in its inactivation [38]. In the normal cell cycle, and after DNA damage has been repaired, WEE1 is phosphorylated by polo-like kinase 1 and subsequently degraded through the ubiquitin proteome system enabling the G2 to M transition [39]. Dephosphorylation of CDK1 by CDC25C phosphatase then leads to the activation of cyclin B/CDK1 complex and initiation of mitosis [37].

In mitosis, proper division of the replicated genome is ensured by a mechanism known as the spindle assembly checkpoint (SAC), which delays the degradation of cyclin B and the anaphase inhibitor securin by prohibiting the ubiquitin ligase anaphase-promoting complex/cyclosome (APC/C) until all chromosomal pairs have bipolar attachment to avoid chromosome separation mistakes [40]. The SAC is activated by malformed or incomplete spindles, and cells that are kept in mitosis by an activated SAC experience apoptosis after extended mitosis [41]. During the normal mitosis, caspase 9 is phosphorylated and suppressed by CDK1 to prevent apoptosis; nevertheless, caspase 9 is eventually dephosphorylated and activated after prolonged mitotic arrest [42]. Furthermore, extended activity of the cyclin B/CDK1 complex has been shown to cause degradation of the anti-apoptotic protein Mcl1, resulting in caspase-dependent death in cells with spindle malformations [43].

## 3. IR-Induced DNA Damage Repair

While ATM and ATR block the progression of cell cycle in response to DNA damage, they also initiate DNA damage repair through phosphorylation of various additional substrates. Base damage and SSBs induced by IR are rapidly and efficiently repaired by base excision repair (BER) while DSBs are repaired by two major mechanisms, non-homologous end-joining (NHEJ) and homologous recombination (HR) [44]. NHEJ is able to join double-stranded DNA ends in all phases of cell cycle whereas HR uses the sister-chromatid as a template and thus operates only in late S and G2. The half-lives of NHEJ and HR are 5–30 min and 2–5 h, respectively, which represent the quick and slow components of DSBs repair [45]. Although NHEJ repair is efficient, it may introduce small deletions or insertions since it catalyzes simple rejoining reactions without sequence homology between DNA ends. NHEJ repairs most direct two-ended IR-induced DSBs and is the main pathway to repair DSBs in the G1 phase, although it is active in all cell-cycle phases except mitosis (Figure 1). The Ku70/Ku80 heterodimer recognizes DSBs and recruits the DNA-dependent protein kinase catalytic subunit (DNA-PKcs) by binding to damaged DNA, which further activates a set of endonucleases and exonucleases such as Artemis, the major enzyme for processing damaged DNA ends [46,47]. Other important NHEJ proteins including X-ray cross-complementing protein 4 (XRCC4), ligase IV and XRCC4-like factor (XLF) are recruited and collectively facilitate the alignment and ligation of DNA ends [48]. In NHEJ, DNA Pol μ and Pol λ fill the DNA gaps before ligation [49,50].

Due to the requirement for widespread end excision and homologous DNA sequences, HR is a rather slow but highly accurate repair mechanism. Although HR plays a minor function in repairing simple IR-induced DSBs, it is essential for the repair of complex DSBs (Figure 1). HR begins with the excision of 5′ DNA ends, followed by the binding of RPA to single-strand 3′ DNA ends [51]. RAD51 replaces RPA with the help of BRCA2, thereby mediating the matching of the homologous sequence to the damaged sequence in the sister chromatid or homologous chromosome and DNA strand invasion [52]. Using the sister chromatid sequence as a template, Pol δ synthesizes error-free DNA and a ligase closes the nick at the freshly extended DNA strand ends [53,54]. Finally, the “Holiday junction” between the two sister chromatids is resolved to separate the two error-free chromatid strands.

Notably, during the DNA repair process, poly (ADP-ribose) polymerase 1 (PARP1) is mainly responsible for detecting SSBs, recruiting DNA repair factors and stabilizing replication forks [55]. PARP1 inhibition has been reported to be integrally fatal when combined with *BRCA1/2* deficiency. The synthetic lethal interaction may be attributed to the HR deficiency caused by *BRCA1/2* mutations, which in turn further impairs DNA repair through PARP1 inhibition, consequently killing tumor cells through a dual effect [56]. Additionally, PARP1 inhibition and BER depletion contribute to the accumulation of SSBs in the S phase, which ultimately generate DSBs after replication fork collapse [57]. Finally, PARP1 is involved in the alternative end-joining (alt-EJ) pathway, which acts as a backup to NHEJ and HR but is much more error-prone [58].

## 4. Targeting Cell-Cycle Checkpoints and DNA Repair Pathways to Enhance Radiosensitivity in CRC

Cellular DDR and DNA repair processes are critical for clonogenic cell survival making these pathways promising targets to overcome radioresistance and improve tumor control [59,60,61]. The targeted proteins involved in the DDR and DNA repair pathways in irradiation of CRC are listed in Supplementary Table S1.

### 4.1. ATM and ATR

ATM and ATR are crucial mediators of the DDR. Owing to their ability to trigger cell-cycle arrest and promote DNA repair through their downstream targets, ATM and ATR inhibitors are considered to improve clinical outcomes of tumor treatment in combination with radiotherapy [62]. ATM is a serine/threonine kinase composed of 3056 amino acids and belongs to the phosphatidylinositol 3-kinase-related protein kinases (PIKKs) family. ATM remains inactive in the form of a homodimer under normal conditions; however, in the presence of IR-induced DSBs, ATM is recruited and activated through intermolecular autophosphorylation and homodimer dissociation, which then triggers the DNA damage checkpoint and facilitates the damaged DNA repair through activation of the NHEJ and HR repair pathways [63]. Somatic ATM mutations are identified in about 20% of CRC patients, usually occurring in the functional domain as heterozygous variants, and loss of ATM expression is also linked to advanced TNM stage and poor 5-year overall survival (OS) in CRC patients [64,65]. Since ATM serves as an apical modulator of DSBs, the administration of ATM inhibitors can effectively increase the radiosensitivity of tumor cells. A preclinical study investigated the potential of the ATM inhibitor KU55933 in radiotherapy [66]. The results showed that KU55933 reduced IR-induced EGFR phosphorylation in CRC cell lines, inhibited tumor cell growth and sensitized tumor cells to IR, as well as decreased the efficiency of HR repair in IR-induced DSBs [66]. Thus, ATM inhibition might serve as an alternative treatment for EGFR inhibitor-resistant CRC. Another novel ATM inhibitor KU59403 with enhanced potency and specificity against ATM radiosensitized CRC cell lines independent of *TP53* status, providing important preclinical data to support the clinical development of ATM inhibitors in the future [67]. In addition, quercetin, a major antioxidant flavonoid, sensitized a CRC cell line to IR in vitro and in vivo, which was associated with a significantly extended presence of IR-induced γH2AX foci and prolonged DNA repair by inhibiting ATM kinase activation [68].

**Figure 2:**
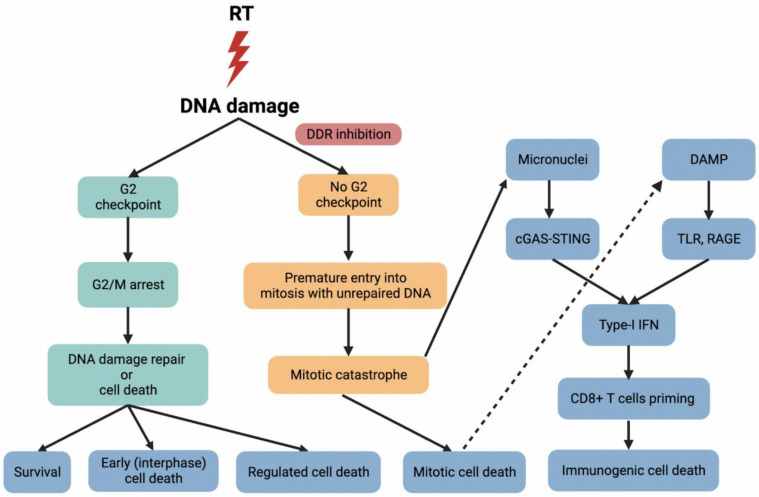
Radiotherapy (RT) causes DNA damage, and DDR inhibitors selectively mediate G2 checkpoint inhibition, allowing cells with unrepaired DNA to prematurely enter into mitosis, ultimately leading to mitotic catastrophe. DNA damage induces the release of damage-associated molecular pattern (DAMP) molecules. For example, HMGB1 is released into the extracellular environment and then mediates potent pro-inflammatory effects by binding to its downstream receptors such as Toll-like receptors (TLR) and receptors for advanced glycation end products (RAGE) to stimulate efficient processing and cross-presentation of tumor antigens from dying cells. Naive T cells differentiate into tumor-specific CD8+ T cells in response to type I interferon (IFN), leading to activation of anti-tumor immune responses and increased immunogenic cell death. On the other hand, DDR inhibition and mitotic death contribute to the formation of micronuclei, which is detected by cyclic GMP-AMP synthase (cGAS) and stimulate production of type I IFN as well as other inflammatory cytokines via the STING pathway, thereby triggering anti-tumor immune responses by CD8+ T cells. For the nomenclature of early cell death, regulated cell death, immunogenic cell death and mitotic catastrophe, please see the review by Galluzzi et al. [74].

ATR consists of 2644 amino acids and is another major member of the PIKKs family. As an apical DDR kinase, ATR maintains genome integrity by phosphorylating multiple enzymes at replication forks, mediated by its downstream target CHK1, resulting in cell-cycle arrest and activation of DNA repair mechanisms [69,70]. ATR inhibition affects the function of DNA damage checkpoints and selectively causes the accumulation of DNA damage in *TP53*-deficient cells, allowing cells with unrepaired DNA to prematurely enter into mitosis and ultimately leading to mitotic catastrophe (Figure 2) [71,72]. One study demonstrated increased radiosensitivity by treatment of a group of CRC cell lines with the selective ATR inhibitor VE-821 [73]. VE-821 not only inhibited hypoxia-induced ATR signaling and HIF-1-mediated signaling, which is a critical regulator of hypoxic response, but also induced DNA damage and influenced replication kinetics. Notably, VE-821 significantly sensitized CRC cells to radiation-induced cell killing under both normal and hypoxic conditions [73].

### 4.2. CHK1 and CHK2

CHK1 is a serine/threonine kinase composed of 476 amino acids. In response to SSBs, CHK1 is phosphorylated by ATR, thereby facilitating the initiation of cell-cycle checkpoints in the S and G2/M phases and DNA damage repair [75]. CHK2 is a serine/threonine kinase consisting of 543 amino acids. When DSBs are recognized by ATM, CHK2 is phosphorylated by ATM at the residue Thr68 and subsequently promotes cell-cycle arrest and DNA repair or apoptosis through phosphorylating its downstream targets, which is faster than transcriptional activation of p21 by p53 [76]. CHK2 has a structurally similar active site to CHK1, there is close crosstalk between them and their activation and substrates also overlap significantly [77]. Because of their roles in maintaining DNA integrity, CHK1 and CHK2 are potential targets for sensitizing tumor cells to radiotherapy [78].

A study identified AZD7762 as a CHK1 inhibitor with equivalent potency against CHK2 in a set of four rectal cancer cell lines. AZD7762 was found to inhibit the IR-induced G2-phase arrest and increase γH2AX foci and apoptosis in vitro. In addition, 5-FU synergized with AZD7762 to further enhance radiosensitivity by AZD7762 [79]. Similar results were found for a selective CHK1 inhibitor Chir-124 in HCT116 colon cancer cells, which eliminated the IR-induced G2/M arrest, resulting in radiosensitization and increased apoptosis of these cells after a dose of 2 Gy. Importantly, these results were not associated with *TP53* mutation status [80]. Another study showed that the CHK1 inhibitor prexasertib (LY2606368) inhibited DNA replication and cell-cycle checkpoint activation in vitro and in vivo, leading to premature entry into mitosis and ultimately apoptosis in *TP53*-deficient CRC stem cells [81]. Notably, this was also the case in cells carrying *KRAS* mutations, a subgroup of CRC that is especially difficult to target and treat [81]. In a study of CRC (HT29) xenografts, AZD7762 combined with radiation significantly delayed tumor growth in comparison to radiation alone, suggesting that AZD7762 could enhance radiosensitivity in vivo [82].

### 4.3. WEE1

WEE1 is a bispecific kinase containing 646 amino acids and can be activated by multiple enzymes including CHK1 in response to the accumulation of DNA damage. The activated WEE1 inactivates the cyclin B/CDK1 complex by phosphorylating CDK1, thereby mediating S- and G2-phase arrest [83]. As a consequence, WEE1 is an important negative regulator of cell cycle and represents an ideal target for the G2/M checkpoint inhibition to potentiate chemoradiotherapy. Indeed, although most anti-cancer treatment strategies aim at causing cell-cycle arrest, the inhibition of WEE1 kinase might release arrested cells with unrepaired damage into mitosis, leading to mitotic catastrophe (Figure 2) [84]. Several preclinical studies of the WEE1 inhibitor adavosertib (AZD1775 or MK-1775) indicated that adavosertib eliminated the G2-phase checkpoint and radiosensitized *TP53*-deficient cells due to mitotic lethality [85,86,87]. However, subsequent studies suggested that raddiosensitization may occur independently of *TP53* status although it may require a deregulated G1/S checkpoint [88]. Therefore, several novel WEE1 inhibitors are undergoing preclinical tests in various cancer types [89].

Only a few studies have been published on WEE1 inhibitors in CRC. However, a previous study showed that MK-1775 increased the cytotoxicity of 5-FU in *TP53*-deficient human colon cancer cells. MK-1775 not only inhibited the phosphorylation of CDK1 at the Tyr15 site, but also abolished the DNA damage checkpoint induced by 5-FU treatment and led to premature mitotic entry through induction of histone H3 phosphorylation [90]. Another study showed that inhibition of WEE1 by small interfering RNA (siRNA) significantly inhibited the proliferation of cancer cells and sensitized *TP53*-mutated colon cancer cell lines HT29 and SW480 to radiotherapy. Moreover, severe DNA damage, suppression of cell viability and apoptosis were observed in these two cell lines treated with MK-1775, indicating the anti-tumor effect of MK-1775 [91]. Based on these findings, WEE1 seems to be a promising target in combination with radiotherapy for CRC harboring *TP53* mutations.

### 4.4. DNA-PKcs/NHEJ

DNA-PKcs, consisting of 4128 amino acids, is also a member of the PIKKs family and a key enzyme participating in the NHEJ repair pathway [92]. Since NHEJ is the primary repair mechanism for IR-induced DSBs, targeting DNA-PKcs with a series of inhibitors can effectively improve the efficacy of radiotherapy. DNA-PKcs specific inhibitors are particularly attractive in combination with radiotherapy as an efficient strategy to improve the prognosis of tumor patients and have been developed as radiosensitizers [93,94]. Studies have evaluated DNA-PKcs inhibitors in other tumor models, but very few studies have been executed for CRC. A previous study used a small biotin-labeled fusion peptide 3 (BTW3) to inhibit the activation of DNA-PKcs in response to DNA damage by specifically targeting its autophosphorylation. BTW3 significantly prolonged IR-induced formation of γH2AX foci and delayed DNA damage repair in human colon cancer RKO cell lines, sensitizing the cells to IR [95]. In another study, a specific DNA-PKcs inhibitor NU7441 radiosensitized *TP53* wild type (LoVo) and *TP53* mutant (SW620) colon cancer cell lines when applied at a non-cytotoxic dose and this effect was not significantly dependent on the *TP53* status of cells. NU7441 not only extended the IR-induced G2/M-phase arrest, but also significantly retarded the disappearance of γH2AX foci, indicating that NU7441 markedly delayed the IR-induced DSBs repair [96]. A recent study demonstrated that the selective DNA-PKcs inhibitor peposertib (M3814) improved the sensitivity of SW837 cell lines to chemoradiotherapy, and reduced the level of phosphorylated DNA-PKcs in SW837 cells when combined with 5-FU and radiation, thus serving as a potent radiosensitizer. Mice with CT26 tumors treated with M3814 in combination with 5-FU and radiation exhibited significantly higher clinical complete response (cCR) but no difference in average tumor size or pCR, which may be due to increased activity of other repair mechanisms that impair the therapeutic effect [97].

### 4.5. HR

As a DNA repair pathway, a minor fraction of DSBs and ICLs are repaired by HR. YU238259, a novel small molecule developed using high-throughput screening, specifically decreased DSBs repair by HR but had no impact on the efficiency of NHEJ [98]. This study showed that YU238259 inhibited IR-induced DSBs repair and increased radiosensitivity in DLD-1 *BRCA*-knockout cell lines and tumor xenografts in nude mice, with the most striking radiosensitization observed in *BRCA2*-deficient cells [98]. Germline mutations in *BRCA* are currently on the rise as a risk factor for CRC due to the significantly increased risk of early-onset CRC in *BRCA* mutation carriers [99]; therefore, YU238259 may have significant clinical potential as a new radiosensitizing agent in *BRCA2*-negative CRC patients.

### 4.6. PARP1

PARP1 is the best-studied PARP enzyme and plays a key role in repairing DNA damage, as well as regulating chromatin decondensation and cell-cycle arrest [55,100]. PARP1 inhibitors developed in recent years have been shown to produce synergistic killing by synthetic lethality in HR-deficient cells. This has been beneficial in the management of *BRCA1/BRCA2*-deficient breast and ovarian cancers and can significantly improve patient prognosis as monotherapy or in combination with conventional therapies [101]. Synthetic lethality is a phenomenon in which the perturbation of a single gene is tolerable for cell survival, while the simultaneous perturbation of multiple genes, for instance through pre-existing loss-of-function mutations, leads to cell death [102]. For example, BER is the primary repair pathway for SSBs in response to IR-induced oxidative damage [103]. BER inhibition results in unrepaired SSBs, which are converted to DSBs upon encountering replication forks [104]. HR deficiency induced by *BRCA1/2* mutations compromises the repair of DSBs, and therefore *BRCA1/2*-mutated tumors exhibit an increased sensitivity to PARP inhibition. However, evidence that PARP1 is intimately associated with CRC came from 91 analyzed tumors, of which PARP1 mRNA overexpression was observed in 64 (70.3%) at the early stages of CRC (65 adenomas and 26 submucosal carcinomas, respectively) [105]. Furthermore, a significant correlation between single nucleotide polymorphisms (SNPs) in the PARP1 gene and CRC risk was identified in a candidate-SNP study of 1176 healthy controls and 310 patients (180 colon and 130 rectal cancer) from the Singapore Chinese Health Study [106]. The above results suggest that PARP1 may have a crucial function in the oncogenesis of CRC, and that PARP inhibitors may be applied in CRC therapy as a potential combination treatment. In response to radiation exposure, PARP1 is rapidly activated and recruited to the damaged DNA, which suggests that combining PARP inhibitors with radiotherapy could produce synergistic effects [107].

The DNA repair activity of PARP1 has been targeted in combination with radiotherapy based on the finding that inhibition of PARP1 may increase the radiosensitivity in cancer cells [108,109]. A previous study found a reduction of 73% in survival in CRC cell lines (LoVo) after treatment with AG14361, a potent small molecule PARP1 inhibitor, when combined with 8 Gy of IR [110]. Under a fractionated radiotherapy regimen, LoVo xenografts showed a tumor growth delay of 19 days, which increased to 37 days in combination with a low dose of AG14361, whereas AG14361 alone failed to retard tumor growth [110]. Another study found that the PARP1 inhibitor ABT-888 (veliparib) treatment significantly increased DSBs and delayed repair in HCT116 and HT29 cells after radiotherapy. In HCT116 xenografts, the tumor growth delay was 7.22 days with radiotherapy alone compared to 11.90 days with 12.5 mg/kg ABT-888 orally administered twice daily accompanied by 2 Gy fractions of radiotherapy [111]. In addition, in an experiment with a subcutaneously implanted osmotic pump combined with IR, ABT-888 was found to have a dose-dependent effect in contrast to IR alone in HCT116 xenografts [112]. Further support for PARP inhibition as a radiosensitizing strategy was found in another study showing that olaparib, which is the inhibitor of PARP1 and PARP2, sensitized DLD-1 cells to radiation in vitro even at a concentration as low as 10 nM. When compared with radiation alone, the induction of γH2AX foci significantly increased with the combined treatment. The topoisomerase I inhibitor camptothecin enhanced the radiosensitizing effect of the PARP inhibitor olaparib, resulting in increased numbers of γH2AX foci and G2/M arrest [113]. According to the evidence presented above, the combination of PARP1 inhibitors and radiotherapy was more successful in vitro and in vivo than any single treatment, supporting the potential value of combining PARP1 inhibition with current radiotherapy regimens.

## 5. Clinical Trials of DDR and DNA Repair Pathway Inhibitors in CRC Patients

Several preclinical trials have investigated the DDR and DNA repair pathways-targeting therapies for CRC as single agents or in combination with conventional chemoradiotherapy. Despite this, clinical research exploiting the DDR and DNA repair defects in CRC patients is still at a very early stage, and none of these therapies has been approved by the Food and Drug Administration (FDA) in CRC patients. In this review, clinical evidence is presented from available studies in the literature about targeting the DDR and DNA repair pathways in CRC.

According to the ClinicalTrials.gov database for clinical trials, there are five completed or ongoing clinical trials investigating the potential role of DDR and DNA repair pathways inhibitors combined with radiotherapy in CRC (Table 1). All completed studies on these inhibitors monotherapy or combined with chemotherapy were either phase I or II trials and the commonly tested agents were CHK1 and PARP1 inhibitors (Table 2). Seven of these studies assessed only CRC patients, while the remaining nineteen studies also included patients with other solid tumors, and the number of CRC patients included in these studies was small, ranging from 2 to 75. Moreover, in the only study evaluating PARP1 inhibitor monotherapy, the objective response rate (ORR) was 0%, while in other studies with PARP1 inhibitors in combination with chemotherapy or radiotherapy, the ORR ranged from 0 to 57%. In terms of therapeutic tolerance, the frequency of grade 3–4 adverse effects (AEs) ranged from 10.3 to 88.9%. Current ongoing trials evaluating the potential of these inhibitors as monotherapy or combined with chemotherapy and/or immunotherapy in CRC are listed in Table 3. 

Based on extensive preclinical data, five PARP inhibitors, olaparib (AZD-2281), veliparib (ABT-888), talazoparib, rucaparib and niraparib, have entered clinical trials for CRC treatment. Olaparib was tested as monotherapy with 33 mCRC patients (20 microsatellite stable (MSS) and 13 microsatellite instability-high (MSI-H)) in a phase II trial (NCT00912743). Results of this trial showed that olaparib was clinically inactive in both MSI-H and MSS mCRC patients [134]. Subsequently, in a phase Ib trial, stage II/III rectal cancer patients received 825 mg/m^2^ capecitabine twice per day and 1.8 Gy radiation per day over about 6 weeks for a total of 50.4 Gy, along with an increased dose of veliparib (20–400 mg) administered orally twice per day (NCT01589419). The maximum tolerated dose (MTD) was not achieved in this study, which ultimately identified 400 mg of veliparib twice per day as the proper dose to be given in a phase II study [115]. A total of 12.5% of patients developed grade 3–4 AEs and 29% achieved pCR, indicating an acceptable safety profile of veriparib in combination with capecitabine and radiotherapy, but preliminary anti-tumor activity required further evaluation in larger studies [115]. Future trials should focus on testing whether PARP inhibitors combined with radiotherapy and/or chemotherapy in CRC patients might increase tumor lethality and improve radiosensitivity with tolerable toxicity. A phase Ib trial evaluating the safety and tolerability of the DNA-PKcs inhibitor M3814 in combination with radiotherapy for locally advanced rectal cancer has been completed (NCT03770689) and showed dose limiting toxicities (DLTs) in five patients. All patients experienced treatment-related adverse events (TRAEs), and grade 3–4 AEs were common (36.8% of all evaluable patients), including hematologic and gastrointestinal toxicity. Another phase I dose-escalation study (NCT03225105) assessed the safety and anti-tumor activity of the ATM inhibitor M3541 (50–300 mg) in combination with fractionated palliative radiotherapy (30 Gy in 10 fractions) in 15 patients with solid tumors (including two CRC patients) [114]. One patient in the 200 mg group experienced two DLTs (urinary tract infection and febrile neutropenia). All patients reported at least one TRAE, and two of them suffered severe adverse events (SAEs), which were not considered to be related to M3541 [114]. Partial or complete responses were confirmed in three patients (20.0%). However, the MTD and recommended phase II dose (RP2D) could not be determined due to the lack of a dose-response relationship [114]. Nevertheless, given the widespread use of radiotherapy and its ability to improve expected outcomes, it makes sense to continue to investigate ATM inhibitors in combination with radiotherapy and assess their potential to enhance radiosensitivity in CRC, where ATM remains an attractive therapeutic target. Actually, the development of a second-generation ATM inhibitor M4076 is underway and the drug has entered the clinical study (NCT04882917) [138]. In addition, the ATR inhibitor AZD6738 combined with radiotherapy (NCT02223923) is currently being studied in phase I clinical trials for the treatment of patients with advanced solid tumors (including CRC) to evaluate the safety and tolerability of AZD6738 as a single agent or in combination with radiotherapy (Table 3).

AZD1775, the only WEE1 inhibitor currently in clinical development, significantly improved progression-free survival (PFS) compared with active monitoring in mCRC with *TP53* and *RAS* mutations in a phase II trial and demonstrated its potential as a well-tolerated treatment for mCRC with *TP53/RAS* mutations [139]. In addition, AZD1775 has shown good tolerability and promising anti-cancer activity when combined with radiotherapy or DNA damaging agents. A phase I trial in pancreatic cancer reported a significantly increased OS using the AZD1775 in combination with radiotherapy and gemcitabine compared to AZD1775 alone [140]. Assuming that the same mechanisms can be translated in CRC and that WEE1 inhibitors have a strong biological rationale for CRC treatment, the positive results of AZD1775, combined with radiotherapy in pancreatic cancer, support the testing and further investigation of this combination therapy in CRC. However, there are no published clinical trial data combining AZD1775 with radiotherapy for the treatment of CRC, and only few clinical trials for CHK1/CHK2 inhibitors in combination with radiotherapy. Currently, WEE1 inhibitors and CHK1/CHK2 inhibitors are under investigation only as single agents or combined with various chemotherapeutic drugs for CRC treatment. Although some anti-tumor activity was indicated, a non-negligible toxicity of the combination of these inhibitors with chemotherapy was noted [116,117,118,119,120,121,122].

Radiation induces genomic DNA damage and, thus, DNA repair inhibitors in combination with radiotherapy might further enhance the efficacy of CRC treatment. However, responses often differ between individual tumors, and some major issues remain with the clinical application of these inhibitors in combination with radiotherapy [141]. First, off-target effects are a main challenge for clinical application of the combination of these inhibitors with radiotherapy; therefore, identifying inhibitors with potential to reduce CRC radioresistance in the DDR pathway should account for both the modulation of radiation-associated signaling and the DDR pathway signaling. Second, to effectively enhance the radiosensitivity of CRC, the focus should be on the safety and tolerability of inhibitor combinations, which is one of the main challenges in current clinical application. Finally, the intrinsic DNA repair capacity of tumor cells is a vital element influencing the therapeutic effect of various inhibitors combinations. Future research into the fundamental molecular mechanisms underlying DNA damage and repair, as well as the identification of biomarkers for successful treatment, should contribute to the development of optimal radiosensitizers and personalized therapies for CRC.

## 6. Combination Therapies

At present, the benefits and drawbacks of using a single DDR and DNA repair pathways inhibitor have been reported in clinical practice. The benefit is that a single inhibitor could take advantage of tumor-specific deficiencies in checkpoint pathways and DNA repair to transform the endogenous DNA damage into the lethal replicative damage in tumor cells, thereby leading to cell death. Additionally, the adverse effects of individual inhibitors would be minimized by crosstalk between normal cells. However, the major limitation of individual inhibitors for tumor therapies is the acquirement of drug resistance. Drug resistance is driven by multiple factors, including increased drug efflux, overexpression of proteins related to DNA repair and suppression of proteins engaged in the apoptotic process [142].

### 6.1. Combination of Different DDR Inhibitors

Given the key role of ATM/ATR, CHK1/CHK2, WEE1, DNA-PKcs in DDR and cell-cycle checkpoint signaling, the combination of one or more of these DDR inhibitors with PARP inhibition would be of great help in inducing replication fork collapse and/or synthetic lethality. AZD7648, a highly selective inhibitor of DNA-PKcs, has been demonstrated in a preclinical study to act as a potent sensitizer of IR-induced DNA damage, thereby promoting tumor cell growth inhibition and apoptosis [143]. A phase I clinical trial (NCT02723864) with veliparib in combination with an ATR inhibitor berzosertib for the treatment of advanced solid tumors reported grade 3–4 AEs in 35.8% of patients; most frequently, bone marrow suppression [137]. In fact, a phase II clinical trial of olaparib combined with AZD6738 and AZD1775 (NCT02576444) is currently ongoing, and the results will soon demonstrate whether the combination of these DDR inhibitors is effective (Table 3).

### 6.2. Combination of DDR Inhibitors with Immunotherapy and Radiotherapy

In recent years, immune checkpoint inhibitors targeting programmed cell death protein 1 (PD-1) have been shown to be effective in mCRC patients with DNA repair defects such as deficient mismatch repair (dMMR) or MSI-H CRC [144]. Nevertheless, anti-PD-1 therapy also has limits, as dMMR/MSI-H CRC accounts for only a small percentage (10–20%) of all CRC [145]. Irradiation with high dose per fraction acts as an immune adjuvant by releasing damage-associated molecular pattern (DAMP) molecules, such as calreticulin, high-mobility-group-box 1 (HMGB1), adenosine triphosphate (ATP) and double-stranded DNA, into the extracellular space [146,147]. Therefore, strategies to increase cell death or switching modes of clonogenic cell death from apoptosis or permanent cell-cycle arrest to more catastrophic modes that release DAMP molecules might not only sensitize cells to radiotherapy but also enhance immunogenic cell death.

After radiotherapy, DAMP molecules are first recognized by pattern recognition receptors; for example, HMGB1 is released into the extracellular environment and then mediates potent pro-inflammatory effects by binding to its downstream receptors such as Toll-like receptors (TLR) and receptor for advanced glycation end products (RAGE) to stimulate efficient processing and cross-presentation of tumor antigens from dying cells [148]. Naive T cells bind to co-stimulatory receptors on dendritic cells and differentiate into tumor-specific CD8+ T cells in response to type I interferon (IFN), leading to activation of anti-tumor immune responses and increased immunogenic cell death (Figure 2) [149]. In a clinical trial of phase IV non-small cell lung cancer, the addition of radiotherapy to immunotherapy improved median progression-free survival (mPFS) from 4.4 to 9.0 months (*p* = 0.045) and median overall survival (mOS) from 8.7 to 19.2 months (*p* = 0.0004). No new safety issues were identified, which provided promising clinical value for the combination of immunotherapy and radiotherapy in CRC, although the optimal immunogenic radiation dose and fractionation regimens remain to be explored [150]. Several recent ongoing trials (NCT04535024, NCT03101475, NCT03507699) combine immunotherapy with radiotherapy to test the ability to increase the immunogenic cell death in CRC [151].

DDR and DNA repair proteins maintain genomic integrity; therefore, DDR and DNA repair inhibitors may increase the tumor mutational burden (TMB) of CRC, which can result in neoantigen production and an increase of anti-tumor T cell activity [152,153,154]. So far, the potential mechanisms of DDR inhibition in combination with immunotherapy have not been fully elucidated, but some interesting facts have been reported. Radiotherapy induces DNA damage and mitochondrial outer membrane permeabilization (MOMP), resulting in the release of mitochondrial DNA (mtDNA) into the cytoplasm. Furthermore, DDR inhibition and mitotic death contribute to the formation of micronuclei. Micronuclei and mtDNA are detected by cyclic GMP-AMP synthase (cGAS) and stimulate production of type I IFN as well as other inflammatory cytokines via the STING pathway, thereby triggering anti-tumor immune responses by CD8+ T cells (Figure 2) [155,156]. A clinical trial of AZD6738 in combination with olaparib, carboplatin or the anti-programmed death ligand 1 (PD-L1) antibody durvalumab in advanced solid tumors including CRC is underway (NCT02264678). Early data showed that the combination therapy of AZD6738 and olaparib exhibited overlapping toxicity profiles, mostly myelosuppression, yet the non-overlapping toxicity of AZD6738 and durvalumab makes the combination of DDR inhibitors and immunotherapy attractive for CRC treatment [157]. In addition, two recent phase I trials are testing the safety and tolerability of AZD1775 combined with durvalumab (NCT02617277) and berzosertib combined with avelumab (NCT04266912) in patients with advanced solid tumors. Moreover, another four ongoing I/II trials (NCT02484404, NCT03851614, NCT03842228, NCT03772561) will investigate the efficacy of olaparib in combination with durvalumab (Table 3).

The non-overlapping toxicity of DDR inhibitors and immunotherapy makes the combination of these drugs with radiotherapy also highly attractive [61,158]. A preclinical study demonstrated that the combination of ATR inhibitor AZD6738 with radiotherapy resulted in enhanced tumor-infiltrating CD8+ T cell activity in CT26 mouse colon cancer cells by blocking radiation-induced PD-L1 expression and significantly reducing the number of tumor-infiltrating Treg. This finding raised the exciting possibility that ATR inhibitors monotherapy could potentiate the cytotoxic effects of radiation while enhancing CD8+ T cell-dependent anti-tumor activity following radiation, resulting in a durable anti-tumor immune response [159]. Until now, although many DDR inhibitors have been developed, only a few of them combined with immunotherapy have achieved clinical study stage, while even fewer have been evaluated in combination with radiotherapy. A phase I clinical trial underway would provide evidence whether M3814 combined with avelumab and radiotherapy is tolerable and effective for treating advanced solid tumors including CRC (NCT03724890). Clearly, a better insight into the interaction between DDR and tumor immunity is required in the future, as well as exploration of the optimal combination of DDR inhibitors with immunotherapy and radiotherapy to improve the clinical outcomes of conventional therapies and increase the therapeutic benefit for CRC patients.

## 7. Conclusions

Radiotherapy is a cornerstone in the treatment of advanced CRC or mCRC, and enhancing radiosensitivity is a promising strategy to improve patients’ prognosis. However, the underlying mechanisms of radioresistance are diverse and, in particular, significantly associated with CRC stem cells. Indeed, targeting key kinases engaged in the DDR and DNA repair pathways, such as ATM/ATR, CHK1/CHK2, WEE1, DNA-PKcs and PARP1, appears to be a promising approach for improving the radiosensitivity to CRC with further insight into the molecular mechanisms underlying IR-induced DNA damage recognition and repair. This review highlights the basic, preclinical and clinical studies investigating DDR and DNA repair pathways inhibitors as a prospective strategy to potentiate the radiation response in CRC by releasing cells with unrepaired damage into mitosis and inducing more catastrophic cell death with increased release of DAMP molecules. Nevertheless, only a few of these inhibitors have been evaluated in combination with radiotherapy in limited preclinical CRC models and clinical trials, indicating that the strategy is still in its infancy in the clinical setting. Importantly, this review also describes the selective chemoradiotherapy sensitization of CRC cells provided by the synthetic lethality of these inhibitors, thus providing additional opportunities to selectively target and increase therapeutic benefits in the future.

Ideally, CRC patients should be stratified based on their genetic background of tumors, as different mutations would display variable degrees of susceptibility to these drugs in comparison to normal tissue. However, the toxicity of drug combinations or absence of patient selection has inhibited the clinical advancement of DDR and DNA repair pathway inhibitors in CRC. This emphasizes the urgency to identify and validate predictive biomarkers of response to these inhibitors in more clinical models to better select and stratify CRC patients, thus allowing the development of more personalized and targeted therapies that reduce the incidence of toxicity and resistance to existing inhibitors.

## Figures and Tables

**Table 1:** Completed or ongoing clinical trials investigating the potential role of DDR and DNA repair pathways inhibitors combined with RT in CRC.

Target	ClinicalTrials.gov Identifier, Authors and Reference	Phase	Status	Disease(s)	Treatment	Primary Outcome Measures	CRC Patients Enrolled	ORR (%)	MTD	Grade 3–4 AEs (%)
ATM	NCT03225105 Waqar [114]	I	Completed	Solid tumors, including CRC	M3541 + RT	DLTs	2	NA	NA	26.7 ^a^
PARP	NCT01589419 Czito [115]	Ib		Locally advanced rectal cancer	Veliparib (ABT-888) + capecitabine + RT	MTD, RP2D	32	9/32 (28.1)	NA	12.5
DNA-PKcs	NCT03770689	Ib		Locally advanced rectal cancer	Peposertib (M3814) + capecitabine + RT	DLTs	19	NA	NA	36.8
	NCT03724890	I	Ongoing	Advanced solid tumors, including CRC	M3814 + avelumab ± RT	DLTs	NA	NA	NA	NA
ATR	NCT02223923	I		Solid tumors, including CRC	Ceralasertib (AZD6738) + RT	MTD	NA	NA	NA	NA

DDR: DNA damage response; CRC: colorectal cancer; AEs: adverse events; ORR: objective response rate; MTD: maximum tolerated dose; NA: not assessable; RT: radiotherapy; DLTs: dose limiting toxicities; RP2D: recommended phase 2 dose. a Data from all patients participating in clinical trials, not only CRC.

**Table 2:** Completed clinical trials investigating the potential role of DDR and DNA repair pathways inhibitors as monotherapy or combined with chemotherapy in CRC.

Target	ClinicalTrials.gov Identifier, Authors and Reference	Phase	Disease(s)	Treatment	CRC Patients Enrolled	ORR (%)	MTD	Grade 3–4 AEs (%)
ATR	NCT02157792 Middleton [116]	I	Advanced solid tumors, including CRC	Berzosertib (M6620, VX-970) + gemcitabne ± cisplatin	22	NA	NA	79.3 ^a^
	NCT02157792 Shapiro [117]	I	Advanced solid tumors, including CRC	Berzosertib (M6620, VX-970) + cisplatin	5	NA	NA	70.0
	NCT02157792 Yap [118]	I	Advanced solid tumors, including CRC	Berzosertib (M6620, VX-970) ± carboplatin	11	NA	90 mg/m^2^	30.4 ^a^
CHK1	NCT02860780 Bendell [119]	I	Advanced/metastatic cancer, including CRC	Prexasertib (LY2606368) + ralimetinib	9	NA	105 mg/m^2^	33.3 ^a^
	NCT02124148 Moore [120]	Ib	Advanced/metastatic cancer, including CRC	Prexasertib (LY2606368) + cetuximab	41	2/41 (4.9)	80 mg/m^2^	53.7
	NCT01115790 Hong [121]	I	Advanced cancer, including CRC	Prexasertib (LY2606368)	9	NA	40 mg/m^2^; 105 mg/m^2^	88.9 ^a^
	NCT02797964 Plummer [122]	I/II	Advanced solid tumors (including CRC), non-hodgkin’s lymphoma	SRA737 (CCT245737)	32	NA	1000 mg/day	44.9 ^a^
	NCT01564251 Italiano [123]	I	Refractory solid tumors (including CRC), or lymphoma	GDC-575 (ARRY-575; RG7741)	4	NA	60 mg/m^2^	49 ^a^
	NCT00413686 Sausville [124]	I	US patients with advanced solid tumors, including CRC	AZD7762 ± gemcitabine	11	NA	30 mg/m^2^	69.0 ^a^
	NCT00937664 Seto [125]	I	Japanese patients with advanced solid tumors, including CRC	AZD7762 ± gemcitabine	5	NA	21 mg/m^2^	60.0 ^a^
	NCT00473616 Ho [126]	I	Advanced solid tumors, including CRC	AZD7762 + irinotecan	29	NA	96 mg/m^2^	10.3 ^a^
WEE1	NCT00648648 Leijen [127]	I	Advanced solid tumors, including CRC	Adavosertib (AZD1775, MK-1775) + gemcitabine + cisplatin or carboplatin	15	1/15 (6.7)	225 mg twice/day; 200 mg twice/day; 175 mg/day	54.7 ^a^
	NCT01748825 Do [128]	I	Advanced solid tumors, including CRC	Adavosertib (AZD1775, MK-1775)	2	NA	225 mg twice/day	56.7 ^a^
	NCT02906059 Cohen [129]	Ib	KRAS, NRAS or BRAF mutated metastatic CRC	Adavosertib (AZD1775, MK-1775) + irinotecan	7	NA	NA	NA
PARP	NCT02033551 Berlin [130]	I	Advanced solid tumors, including CRC	Veliparib (ABT-888) + FOLFIRI	10	2/10 (20.0)	NA	38.0 ^a^
	NCT00535353 Chen [131]	I	Advanced or metastatic CRC	Olaparib (AZD-2281) + irinotecan	25	0/25 (0.0)	NA	76.0 ^b^
	NCT02305758 Gorbunova [132]	II	Untreated metastatic CRC	Veliparib (ABT-888) + FOLFIRI ± bevacizumab	65	37/65 (57)	NA	59.0 ^b^
	NCT00553189 Kummar [133]	I	Solid tumors (including CRC) and lymphomas	Veliparib (ABT-888) + topotecan	5	0/5 (0.0)	10 mg twice/day	70.0 ^ab^
	NCT00912743 Leichmann [134]	II	Chemorefractory metastatic CRC	Olaparib (AZD-2281)	33	0/33 (0.0)	NA	48.5
	NCT01051596 Pishvaian [135]	II	Heavily pretreated metastatic CRC	Veliparib (ABT-888) + temozolomide	75	2/75 (2.7)	NA	18.7
	NCT00516438 Samol [136]	I	Advanced solid tumors, including CRC	Olaparib (AZD-2281) + topotecan	8	0/8 (0.0)	100 mg twice/day	47.4 ^a^
	NCT03875313	Ib/II	Solid tumors, including CRC	Talazoparib + CB-839 (Telaglenastat)	4	0/4 (0.0)	NA	18.2 ^a^
PARP, ATR	NCT02723864 Smith [137]	I	Refractory solid tumors, including CRC	veliparib (ABT-888) + berzosertib (M6620, VX-970) + cisplatin	3	NA	NA	35.8 ^a^

DDR: DNA damage response; CRC: colorectal cancer; AEs: adverse events; ORR: objective response rate; MTD: maximum tolerated dose; NA: not assessable; RT: radiotherapy. ^a^ Data from all patients participating in clinical trials, not only CRC. ^b^ No data are available on the number of patients underwent grade 3–4 AEs; the data in the table referred to the incidence of neutropenia, which was the mostly frequent grade 3–4 AEs.

**Table 3:** Ongoing clinical trials evaluating the potential role of DDR and DNA repair pathways inhibitors monotherapy or combined with chemotherapy and/or immunotherapy in CRC.

Target	ClinicalTrials.gov Identifier	Phase	Disease(s)	Treatment	Primary Outcome Measures
ATM	NCT02588105	I	Advanced solid tumors, including CRC	AZD0156 ± olaparib/FOLFIRI	TRAEs
ATR	NCT03188965	I	Advanced solid tumors, including CRC, and lymphomas	Elimusertib (BAY 1895344)	MTD, RP2D, DLTs, TEAEs
	NCT04535401	I	Advanced or metastatic CRC and gastric/gastroesophageal cancers	Elimusertib (BAY 1895344) + FOLFIRI	MTD
	NCT04704661	I/Ib	Advanced solid tumors, including CRC that have a change (mutation) in the HER2 gene or protein	Ceralasertib (AZD6738) + trastuzumab deruxtecan (DS-8201a)	TRAEs, RP2D, PD profile
	NCT02595931	I	Metastatic or unresectable solid tumors, including CRC	Berzosertib (M6620, VX-970) + irinotecan	MTD, RP2D
	NCT04266912	I/II	DDR deficient metastatic or unresectable solid tumors, including CRC	Berzosertib (M6620, VX-970) + avelumab	AEs, SAEs, DLTs, MTD
CHK1	NCT02632448	Ib/IIa	Solid tumors, including CRC	LY2880070 ± gemcitabine	MTD
WEE1	NCT02465060	II	Advanced refractory solid tumors (including CRC), lymphomas, or multiple myeloma	Adavosertib (AZD1775) + targeted therapy according to mutational status	ORR
	NCT04158336	I/II	Solid tumors, including CRC	ZN-c3	MTD, RP2D, ORR
	NCT02617277	I	Advanced solid tumors, including CRC	AZD1775 + durvalumab	DLTs
PARP	NCT02484404	I/II	Ovarian, triple negative breast, lung, prostate, CRC	Durvalumab (MEDI4736) + olaparib ± cediranib	OR, RP2D
	NCT03851614 (DAPPER)	II	Mismatch repair proficient CRC, pancreatic adenocarcinoma, leiomyosarcoma	Durvalumab (MEDI4736) + olaparib + cediranib	Changes in genomic and immune biomarkers
	NCT04171700 (LODESTAR)	II	Solid tumors, including CRC	Rucaparib	ORR
	NCT03251612	II	Metastatic CRC	Olaparib + therapy based on sensitivity analysis	PFS
	NCT03983993 (NIPAVect)	II	Advanced or metastatic CRC	Panitumumab + niraparib	CBR
	NCT03337087	I/II	Metastatic pancreatic, CRC, gastroesophageal, or biliary cancer	Liposomal irinotecan + leucovorin calcium + fluorouracil + rucaparib	MTD, OR, BRR
	NCT04166435	II	MGMT hypermethylated CRC	Temozolomide + olaparib	ORR
	NCT04456699	III	Unresectable or metastatic CRC	Olaparib ± bevacizumab + 5-FU	PFS
	NCT04511039	I	CRC or gastroesophageal cancer	Trifluridine/Tipiracil + talazoparib	AEs, MTD, RP2D
	NCT03842228	Ib	Advanced solid tumors, including CRC	Olaparib + durvalumab + copanlisib (PI3K inhibitor)	MTD
	NCT04123366	II	HRRm and HRD-positive advanced solid tumors, including CRC	Olaparib + pembrolizumab	ORR
	NCT03772561	I	Advanced solid tumors, including CRC	Olaparib + durvalumab + AZD5363 (AKT inhibitor)	ORR
PARP, ATR	NCT02264678	I	Advanced solid tumors, including CRC	Olaparib + ceralasertib (AZD6738) + durvalumab + carboplatin	AEs, SAEs, ECG
	NCT04497116	I/IIa	Advanced solid tumors, including CRC	RP-3500 (ATR inhibitor) ± talazoparib±gemcitabine	MTD, DLTs
PARP, ATR, WEE1	NCT02576444	II	Advanced solid tumors, including CRC	Olaparib + AZD6738 + AZD1775 + AZD5363	ORR

CRC: colorectal cancer; MTD: maximum tolerated dose; TRAEs: treatment-related adverse events; RP2D: recommended phase 2 dose; DLTs: dose limiting toxicities; PD: pharmacodynamics; RT: radiotherapy; ORR: objective response rate; OR: objective response; PFS: progression-free survival; CBR: clinical benefit rate; BRR: best response rate; 5-FU: 5-fluorouracil; PI3K: phosphatidylinositol 3-kinase; HRRm: homologous recombination repair mutation; HRD: homologous recombination deficiency; AEs: adverse events; SAEs: severe adverse events; DDR: DNA damage response; ECG: electrocardiogram.

## Supplementary Materials

The following supporting information can be downloaded at: https://www.mdpi.com/article/10.3390/cancers14194874/s1, Table S1: Targeted proteins involved in the DDR and DNA repair pathways in response to IR.

## References

[1] Sung H., Ferlay J., Siegel R.L., Laversanne M., Soerjomataram I., Jemal A., Bray F. (2021). Global Cancer Statistics 2020: GLOBOCAN Estimates of Incidence and Mortality Worldwide for 36 Cancers in 185 Countries. CA Cancer J. Clin..

[2] Arnold M., Sierra M.S., Laversanne M., Soerjomataram I., Jemal A., Bray F. (2017). Global patterns and trends in colorectal cancer incidence and mortality. Gut.

[3] Miller K.D., Nogueira L., Devasia T., Mariotto A.B., Yabroff K.R., Jemal A., Kramer J., Siegel R.L. (2022). Cancer treatment and survivorship statistics, 2022. CA Cancer J. Clin..

[4] Van Cutsem E., Cervantes A., Adam R., Sobrero A., Van Krieken J.H., Aderka D., Aranda Aguilar E., Bardelli A., Benson A., Bodoky G. (2016). ESMO consensus guidelines for the management of patients with metastatic colorectal cancer. Ann. Oncol. Off. J. Eur. Soc. Med. Oncol..

[5] Yoshino T., Arnold D., Taniguchi H., Pentheroudakis G., Yamazaki K., Xu R.H., Kim T.W., Ismail F., Tan I.B., Yeh K.H. (2018). Pan-Asian adapted ESMO consensus guidelines for the management of patients with metastatic colorectal cancer: A JSMO-ESMO initiative endorsed by CSCO, KACO, MOS, SSO and TOS. Ann. Oncol. Off. J. Eur. Soc. Med. Oncol..

[6] Cercek A., Roxburgh C.S.D., Strombom P., Smith J.J., Temple L.K.F., Nash G.M., Guillem J.G., Paty P.B., Yaeger R., Stadler Z.K. (2018). Adoption of Total Neoadjuvant Therapy for Locally Advanced Rectal Cancer. JAMA Oncol..

[7] Patel P.A. (2011). Evolution of 5-fluorouracil-based chemoradiation in the management of rectal cancer. Anti-Cancer Drugs.

[8] Toulany M., Rodemann H.P. (2010). Membrane receptor signaling and control of DNA repair after exposure to ionizing radiation. Nuklearmedizin. Nucl. Med..

[9] Roy S., Trinchieri G. (2017). Microbiota: A key orchestrator of cancer therapy. Nat. Rev. Cancer.

[10] Barker H.E., Paget J.T., Khan A.A., Harrington K.J. (2015). The tumour microenvironment after radiotherapy: Mechanisms of resistance and recurrence. Nat. Rev. Cancer.

[11] Baumann M., Krause M., Hill R. (2008). Exploring the role of cancer stem cells in radioresistance. Nat. Rev. Cancer.

[12] Luo C.W., Wang J.Y., Hung W.C., Peng G., Tsai Y.L., Chang T.M., Chai C.Y., Lin C.H., Pan M.R. (2017). G9a governs colon cancer stem cell phenotype and chemoradioresistance through PP2A-RPA axis-mediated DNA damage response. Radiother. Oncol. J. Eur. Soc. Ther. Radiol. Oncol..

[13] Schulz A., Meyer F., Dubrovska A., Borgmann K. (2019). Cancer Stem Cells and Radioresistance: DNA Repair and Beyond. Cancers.

[14] Anuja K., Chowdhury A.R., Saha A., Roy S., Rath A.K., Kar M., Banerjee B. (2019). Radiation-induced DNA damage response and resistance in colorectal cancer stem-like cells. Int. J. Radiat. Biol..

[15] Roos W.P., Thomas A.D., Kaina B. (2016). DNA damage and the balance between survival and death in cancer biology. Nat. Rev. Cancer.

[16] Maréchal A., Zou L. (2013). DNA damage sensing by the ATM and ATR kinases. Cold Spring Harb. Perspect. Biol..

[17] Shibata A., Jeggo P.A. (2021). ATM’s Role in the Repair of DNA Double-Strand Breaks. Genes.

[18] García-Santisteban I., Llopis A., Krenning L., Vallejo-Rodríguez J., van den Broek B., Zubiaga A.M., Medema R.H. (2021). Sustained CHK2 activity, but not ATM activity, is critical to maintain a G1 arrest after DNA damage in untransformed cells. BMC Biol..

[19] Mian E., Wiesmüller L. (2017). Phenotypic Analysis of ATM Protein Kinase in DNA Double-Strand Break Formation and Repair. Methods Mol. Biol..

[20] Ma M., Rodriguez A., Sugimoto K. (2020). Activation of ATR-related protein kinase upon DNA damage recognition. Curr. Genet..

[21] Ditano J.P., Sakurikar N., Eastman A. (2021). Activation of CDC25A phosphatase is limited by CDK2/cyclin A-mediated feedback inhibition. Cell Cycle.

[22] Neizer-Ashun F., Bhattacharya R. (2021). Reality CHEK: Understanding the biology and clinical potential of CHK1. Cancer Lett..

[23] Smith H.L., Southgate H., Tweddle D.A., Curtin N.J. (2020). DNA damage checkpoint kinases in cancer. Expert Rev. Mol. Med..

[24] Rubin S.M., Sage J., Skotheim J.M. (2020). Integrating Old and New Paradigms of G1/S Control. Mol. Cell.

[25] Peng G., Cao R.B., Li Y.H., Zou Z.W., Huang J., Ding Q. (2014). Alterations of cell cycle control proteins SHP-1/2, p16, CDK4 and cyclin D1 in radioresistant nasopharyngeal carcinoma cells. Mol. Med. Rep..

[26] Levine A.J. (2020). p53: 800 million years of evolution and 40 years of discovery. Nat. Rev. Cancer.

[27] Hernández-Monge J., Rousset-Roman A.B., Medina-Medina I., Olivares-Illana V. (2016). Dual function of MDM2 and MDMX toward the tumor suppressors p53 and RB. Genes Cancer.

[28] Li S.J., Liang X.Y., Li H.J., Li W., Zhou L., Chen H.Q., Ye S.G., Yu D.H., Cui J.W. (2017). Low-dose irradiation promotes proliferation of the human breast cancer MDA-MB-231 cells through accumulation of mutant P53. Int. J. Oncol..

[29] Cheng Q., Chen J. (2010). Mechanism of p53 stabilization by ATM after DNA damage. Cell Cycle.

[30] Cheng Q., Cross B., Li B., Chen L., Li Z., Chen J. (2011). Regulation of MDM2 E3 ligase activity by phosphorylation after DNA damage. Mol. Cell. Biol..

[31] Mansilla S.F., de la Vega M.B., Calzetta N.L., Siri S.O., Gottifredi V. (2020). CDK-Independent and PCNA-Dependent Functions of p21 in DNA Replication. Genes.

[32] Chen S., Zhou Q., Guo Z., Wang Y., Wang L., Liu X., Lu M., Ju L., Xiao Y., Wang X. (2020). Inhibition of MELK produces potential anti-tumour effects in bladder cancer by inducing G1/S cell cycle arrest via the ATM/CHK2/p53 pathway. J. Cell. Mol. Med..

[33] (2012). Comprehensive molecular characterization of human colon and rectal cancer. Nature.

[34] Errico A., Costanzo V. (2012). Mechanisms of replication fork protection: A safeguard for genome stability. Crit. Rev. Biochem. Mol. Biol..

[35] Saldivar J.C., Hamperl S., Bocek M.J., Chung M., Bass T.E., Cisneros-Soberanis F., Samejima K., Xie L., Paulson J.R., Earnshaw W.C. (2018). An intrinsic S/G(2) checkpoint enforced by ATR. Science.

[36] Sadeghi H., Golalipour M., Yamchi A., Farazmandfar T., Shahbazi M. (2019). CDC25A pathway toward tumorigenesis: Molecular targets of CDC25A in cell-cycle regulation. J. Cell. Biochem..

[37] Liu K., Zheng M., Lu R., Du J., Zhao Q., Li Z., Li Y., Zhang S. (2020). The role of CDC25C in cell cycle regulation and clinical cancer therapy: A systematic review. Cancer Cell Int..

[38] Ghelli Luserna di Rorà A., Cerchione C., Martinelli G., Simonetti G. (2020). A WEE1 family business: Regulation of mitosis, cancer progression, and therapeutic target. J. Hematol. Oncol..

[39] Schmidt M., Rohe A., Platzer C., Najjar A., Erdmann F., Sippl W. (2017). Regulation of G2/M Transition by Inhibition of WEE1 and PKMYT1 Kinases. Molecules.

[40] Stukenberg P.T., Burke D.J. (2015). Connecting the microtubule attachment status of each kinetochore to cell cycle arrest through the spindle assembly checkpoint. Chromosoma.

[41] Rieder C.L., Maiato H. (2004). Stuck in division or passing through: What happens when cells cannot satisfy the spindle assembly checkpoint. Dev. Cell.

[42] Allan L.A., Clarke P.R. (2007). Phosphorylation of caspase-9 by CDK1/cyclin B1 protects mitotic cells against apoptosis. Mol. Cell.

[43] Harley M.E., Allan L.A., Sanderson H.S., Clarke P.R. (2010). Phosphorylation of Mcl-1 by CDK1-cyclin B1 initiates its Cdc20-dependent destruction during mitotic arrest. EMBO J..

[44] Brown J.S., O’Carrigan B., Jackson S.P., Yap T.A. (2017). Targeting DNA Repair in Cancer: Beyond PARP Inhibitors. Cancer Discov..

[45] Jeggo P.A., Geuting V., Löbrich M. (2011). The role of homologous recombination in radiation-induced double-strand break repair. Radiother. Oncol. J. Eur. Soc. Ther. Radiol. Oncol..

[46] Rouhani M. (2019). Modeling the interplay between DNA-PK, Artemis, and ATM in non-homologous end-joining repair in G1 phase of the cell cycle. J. Biol. Phys..

[47] Chang H.H.Y., Pannunzio N.R., Adachi N., Lieber M.R. (2017). Non-homologous DNA end joining and alternative pathways to double-strand break repair. Nat. Rev. Mol. Cell Biol..

[48] Ochi T., Blackford A.N., Coates J., Jhujh S., Mehmood S., Tamura N., Travers J., Wu Q., Draviam V.M., Robinson C.V. (2015). DNA repair. PAXX, a paralog of XRCC4 and XLF, interacts with Ku to promote DNA double-strand break repair. Science.

[49] Andrade P., Martín M.J., Juárez R., López de Saro F., Blanco L. (2009). Limited terminal transferase in human DNA polymerase mu defines the required balance between accuracy and efficiency in NHEJ. Proc. Natl. Acad. Sci. USA.

[50] Capp J.P., Boudsocq F., Bertrand P., Laroche-Clary A., Pourquier P., Lopez B.S., Cazaux C., Hoffmann J.S., Canitrot Y. (2006). The DNA polymerase lambda is required for the repair of non-compatible DNA double strand breaks by NHEJ in mammalian cells. Nucleic Acids Res..

[51] Huang F., Goyal N., Sullivan K., Hanamshet K., Patel M., Mazina O.M., Wang C.X., An W.F., Spoonamore J., Metkar S. (2016). Targeting BRCA1- and BRCA2-deficient cells with RAD52 small molecule inhibitors. Nucleic Acids Res..

[52] Piazza A., Heyer W.D. (2019). Homologous Recombination and the Formation of Complex Genomic Rearrangements. Trends Cell Biol..

[53] Li J., Holzschu D.L., Sugiyama T. (2013). PCNA is efficiently loaded on the DNA recombination intermediate to modulate polymerase δ, η, and ζ activities. Proc. Natl. Acad. Sci. USA.

[54] Maloisel L., Fabre F., Gangloff S. (2008). DNA polymerase delta is preferentially recruited during homologous recombination to promote heteroduplex DNA extension. Mol. Cell. Biol..

[55] Javle M., Curtin N.J. (2011). The role of PARP in DNA repair and its therapeutic exploitation. Br. J. Cancer.

[56] Giovannini S., Weller M.C., Repmann S., Moch H., Jiricny J. (2019). Synthetic lethality between BRCA1 deficiency and poly(ADP-ribose) polymerase inhibition is modulated by processing of endogenous oxidative DNA damage. Nucleic Acids Res..

[57] Beck C., Robert I., Reina-San-Martin B., Schreiber V., Dantzer F. (2014). Poly(ADP-ribose) polymerases in double-strand break repair: Focus on PARP1, PARP2 and PARP3. Exp. Cell Res..

[58] Iliakis G., Murmann T., Soni A. (2015). Alternative end-joining repair pathways are the ultimate backup for abrogated classical non-homologous end-joining and homologous recombination repair: Implications for the formation of chromosome translocations. Mutat. Res. Genet. Toxicol. Environ. Mutagen..

[59] Santivasi W.L., Xia F. (2014). Ionizing radiation-induced DNA damage, response, and repair. Antioxid. Redox Signal..

[60] Morgan M.A., Lawrence T.S. (2015). Molecular Pathways: Overcoming Radiation Resistance by Targeting DNA Damage Response Pathways. Clin. Cancer Res. Off. J. Am. Assoc. Cancer Res..

[61] Pilié P.G., Tang C., Mills G.B., Yap T.A. (2019). State-of-the-art strategies for targeting the DNA damage response in cancer. Nat. Rev. Clin. Oncol..

[62] Weber A.M., Ryan A.J. (2015). ATM and ATR as therapeutic targets in cancer. Pharmacol. Ther..

[63] Wang X., Chu H., Lv M., Zhang Z., Qiu S., Liu H., Shen X., Wang W., Cai G. (2016). Structure of the intact ATM/Tel1 kinase. Nat. Commun..

[64] Jette N.R., Kumar M., Radhamani S., Arthur G., Goutam S., Yip S., Kolinsky M., Williams G.J., Bose P., Lees-Miller S.P. (2020). ATM-Deficient Cancers Provide New Opportunities for Precision Oncology. Cancers.

[65] Lu Y., Gao J., Lu Y. (2014). Downregulated Ku70 and ATM associated to poor prognosis in colorectal cancer among Chinese patients. OncoTargets Ther..

[66] Tang S., Li Z., Yang L., Shen L., Wang Y. (2020). A potential new role of ATM inhibitor in radiotherapy: Suppressing ionizing Radiation-Activated EGFR. Int. J. Radiat. Biol..

[67] Batey M.A., Zhao Y., Kyle S., Richardson C., Slade A., Martin N.M., Lau A., Newell D.R., Curtin N.J. (2013). Preclinical evaluation of a novel ATM inhibitor, KU59403, in vitro and in vivo in p53 functional and dysfunctional models of human cancer. Mol. Cancer Ther..

[68] Lin C., Yu Y., Zhao H.G., Yang A., Yan H., Cui Y. (2012). Combination of quercetin with radiotherapy enhances tumor radiosensitivity in vitro and in vivo. Radiother. Oncol. J. Eur. Soc. Ther. Radiol. Oncol..

[69] Saldivar J.C., Cortez D., Cimprich K.A. (2017). The essential kinase ATR: Ensuring faithful duplication of a challenging genome. Nat. Rev. Mol. Cell Biol..

[70] Flynn R.L., Zou L. (2011). ATR: A master conductor of cellular responses to DNA replication stress. Trends Biochem. Sci..

[71] Kwok M., Davies N., Agathanggelou A., Smith E., Oldreive C., Petermann E., Stewart G., Brown J., Lau A., Pratt G. (2016). ATR inhibition induces synthetic lethality and overcomes chemoresistance in TP53- or ATM-defective chronic lymphocytic leukemia cells. Blood.

[72] Kwok M., Davies N., Agathanggelou A., Smith E., Petermann E., Yates E., Brown J., Lau A., Stankovic T. (2015). Synthetic lethality in chronic lymphocytic leukaemia with DNA damage response defects by targeting the ATR pathway. Lancet.

[73] Pires I.M., Olcina M.M., Anbalagan S., Pollard J.R., Reaper P.M., Charlton P.A., McKenna W.G., Hammond E.M. (2012). Targeting radiation-resistant hypoxic tumour cells through ATR inhibition. Br. J. Cancer.

[74] Galluzzi L., Vitale I., Aaronson S.A., Abrams J.M., Adam D., Agostinis P., Alnemri E.S., Altucci L., Amelio I., Andrews D.W. (2018). Molecular mechanisms of cell death: Recommendations of the Nomenclature Committee on Cell Death 2018. Cell Death Differ..

[75] González Besteiro M.A., Gottifredi V. (2015). The fork and the kinase: A DNA replication tale from a CHK1 perspective. Mutat. Res. Rev. Mutat. Res..

[76] Zannini L., Delia D., Buscemi G. (2014). CHK2 kinase in the DNA damage response and beyond. J. Mol. Cell Biol..

[77] Matthews T.P., Jones A.M., Collins I. (2013). Structure-based design, discovery and development of checkpoint kinase inhibitors as potential anticancer therapies. Expert Opin. Drug Discov..

[78] Chen Z., Xiao Z., Gu W.Z., Xue J., Bui M.H., Kovar P., Li G., Wang G., Tao Z.F., Tong Y. (2006). Selective Chk1 inhibitors differentially sensitize p53-deficient cancer cells to cancer therapeutics. Int. J. Cancer.

[79] Kleiman L.B., Krebs A.M., Kim S.Y., Hong T.S., Haigis K.M. (2013). Comparative analysis of radiosensitizers for K-RAS mutant rectal cancers. PLoS ONE.

[80] Tao Y., Leteur C., Yang C., Zhang P., Castedo M., Pierré A., Golsteyn R.M., Bourhis J., Kroemer G., Deutsch E. (2009). Radiosensitization by Chir-124, a selective CHK1 inhibitor: Effects of p53 and cell cycle checkpoints. Cell Cycle.

[81] Manic G., Signore M., Sistigu A., Russo G., Corradi F., Siteni S., Musella M., Vitale S., De Angelis M.L., Pallocca M. (2018). CHK1-targeted therapy to deplete DNA replication-stressed, p53-deficient, hyperdiploid colorectal cancer stem cells. Gut.

[82] Mitchell J.B., Choudhuri R., Fabre K., Sowers A.L., Citrin D., Zabludoff S.D., Cook J.A. (2010). In vitro and in vivo radiation sensitization of human tumor cells by a novel checkpoint kinase inhibitor, AZD7762. Clin. Cancer Res. Off. J. Am. Assoc. Cancer Res..

[83] Elbæk C.R., Petrosius V., Sørensen C.S. (2020). WEE1 kinase limits CDK activities to safeguard DNA replication and mitotic entry. Mutat. Res..

[84] Mir S.E., De Witt Hamer P.C., Krawczyk P.M., Balaj L., Claes A., Niers J.M., Van Tilborg A.A., Zwinderman A.H., Geerts D., Kaspers G.J. (2010). In silico analysis of kinase expression identifies WEE1 as a gatekeeper against mitotic catastrophe in glioblastoma. Cancer Cell.

[85] Hirai H., Iwasawa Y., Okada M., Arai T., Nishibata T., Kobayashi M., Kimura T., Kaneko N., Ohtani J., Yamanaka K. (2009). Small-molecule inhibition of Wee1 kinase by MK-1775 selectively sensitizes p53-deficient tumor cells to DNA-damaging agents. Mol. Cancer Ther..

[86] Aarts M., Sharpe R., Garcia-Murillas I., Gevensleben H., Hurd M.S., Shumway S.D., Toniatti C., Ashworth A., Turner N.C. (2012). Forced mitotic entry of S-phase cells as a therapeutic strategy induced by inhibition of WEE1. Cancer Discov..

[87] Bridges K.A., Hirai H., Buser C.A., Brooks C., Liu H., Buchholz T.A., Molkentine J.M., Mason K.A., Meyn R.E. (2011). MK-1775, a novel Wee1 kinase inhibitor, radiosensitizes p53-defective human tumor cells. Clin. Cancer Res..

[88] Bukhari A.B., Chan G.K., Gamper A.M. (2022). Targeting the DNA Damage Response for Cancer Therapy by Inhibiting the Kinase Wee1. Front. Oncol..

[89] Vakili-Samiani S., Khanghah O.J., Gholipour E., Najafi F., Zeinalzadeh E., Samadi P., Sarvarian P., Pourvahdani S., Kelaye S.K., Hamblin M.R. (2022). Cell cycle involvement in cancer therapy; WEE1 kinase, a potential target as therapeutic strategy. Mutat. Res..

[90] Hirai H., Arai T., Okada M., Nishibata T., Kobayashi M., Sakai N., Imagaki K., Ohtani J., Sakai T., Yoshizumi T. (2010). MK-1775, a small molecule Wee1 inhibitor, enhances anti-tumor efficacy of various DNA-damaging agents, including 5-fluorouracil. Cancer Biol. Ther..

[91] Yin Y., Shen Q., Tao R., Chang W., Li R., Xie G., Liu W., Zhang P., Tao K. (2018). Wee1 inhibition can suppress tumor proliferation and sensitize p53 mutant colonic cancer cells to the anticancer effect of irinotecan. Mol. Med. Rep..

[92] Yue X., Bai C., Xie D., Ma T., Zhou P.K. (2020). DNA-PKcs: A Multi-Faceted Player in DNA Damage Response. Front. Genet..

[93] Ismail I.H., Mårtensson S., Moshinsky D., Rice A., Tang C., Howlett A., McMahon G., Hammarsten O. (2004). SU11752 inhibits the DNA-dependent protein kinase and DNA double-strand break repair resulting in ionizing radiation sensitization. Oncogene.

[94] Willoughby C.E., Jiang Y., Thomas H.D., Willmore E., Kyle S., Wittner A., Phillips N., Zhao Y., Tudhope S.J., Prendergast L. (2020). Selective DNA-PKcs inhibition extends the therapeutic index of localized radiotherapy and chemotherapy. J. Clin. Investig..

[95] Sun X., Yang C., Liu H., Wang Q., Wu S.X., Li X., Xie T., Brinkman K.L., Teh B.S., Butler E.B. (2012). Identification and characterization of a small inhibitory peptide that can target DNA-PKcs autophosphorylation and increase tumor radiosensitivity. Int. J. Radiat. Oncol. Biol. Phys..

[96] Zhao Y., Thomas H.D., Batey M.A., Cowell I.G., Richardson C.J., Griffin R.J., Calvert A.H., Newell D.R., Smith G.C., Curtin N.J. (2006). Preclinical evaluation of a potent novel DNA-dependent protein kinase inhibitor NU7441. Cancer Res..

[97] Smithson M., Irwin R.K., Williams G., McLeod M.C., Choi E.K., Ganguly A., Pepple A., Cho C.S., Willey C.D., Leopold J. (2022). Inhibition of DNA-PK may improve response to neoadjuvant chemoradiotherapy in rectal cancer. Neoplasia.

[98] Stachelek G.C., Peterson-Roth E., Liu Y., Fernandez R.J., Pike L.R., Qian J.M., Abriola L., Hoyer D., Hungerford W., Merkel J. (2015). YU238259 Is a Novel Inhibitor of Homology-Dependent DNA Repair That Exhibits Synthetic Lethality and Radiosensitization in Repair-Deficient Tumors. Mol. Cancer Res. MCR.

[99] Oh M., McBride A., Yun S., Bhattacharjee S., Slack M., Martin J.R., Jeter J., Abraham I. (2018). BRCA1 and BRCA2 Gene Mutations and Colorectal Cancer Risk: Systematic Review and Meta-analysis. J. Natl. Cancer Inst..

[100] Kamaletdinova T., Fanaei-Kahrani Z., Wang Z.Q. (2019). The Enigmatic Function of PARP1: From PARylation Activity to PAR Readers. Cells.

[101] Poggio F., Bruzzone M., Ceppi M., Conte B., Martel S., Maurer C., Tagliamento M., Viglietti G., Del Mastro L., de Azambuja E. (2018). Single-agent PARP inhibitors for the treatment of patients with BRCA-mutated HER2-negative metastatic breast cancer: A systematic review and meta-analysis. ESMO Open.

[102] O’Neil N.J., Bailey M.L., Hieter P. (2017). Synthetic lethality and cancer. Nat. Rev. Genet..

[103] Biau J., Chautard E., Verrelle P., Dutreix M. (2019). Altering DNA Repair to Improve Radiation Therapy: Specific and Multiple Pathway Targeting. Front. Oncol..

[104] Kuzminov A. (2001). Single-strand interruptions in replicating chromosomes cause double-strand breaks. Proc. Natl. Acad. Sci. USA.

[105] Nosho K., Yamamoto H., Mikami M., Taniguchi H., Takahashi T., Adachi Y., Imamura A., Imai K., Shinomura Y. (2006). Overexpression of poly(ADP-ribose) polymerase-1 (PARP-1) in the early stage of colorectal carcinogenesis. Eur. J. Cancer.

[106] Stern M.C., Conti D.V., Siegmund K.D., Corral R., Yuan J.M., Koh W.P., Yu M.C. (2007). DNA repair single-nucleotide polymorphisms in colorectal cancer and their role as modifiers of the effect of cigarette smoking and alcohol in the Singapore Chinese Health Study. Cancer Epidemiol. Biomark. Prev. Publ. Am. Assoc. Cancer Res. Cosponsored Am. Soc. Prev. Oncol..

[107] Haince J.F., McDonald D., Rodrigue A., Déry U., Masson J.Y., Hendzel M.J., Poirier G.G. (2008). PARP1-dependent kinetics of recruitment of MRE11 and NBS1 proteins to multiple DNA damage sites. J. Biol. Chem..

[108] Vormoor B., Schlosser Y.T., Blair H., Sharma A., Wilkinson S., Newell D.R., Curtin N. (2017). Sensitizing Ewing sarcoma to chemo- and radiotherapy by inhibition of the DNA-repair enzymes DNA protein kinase (DNA-PK) and poly-ADP-ribose polymerase (PARP) 1/2. Oncotarget.

[109] Hirai T., Saito S., Fujimori H., Matsushita K., Nishio T., Okayasu R., Masutani M. (2016). Radiosensitization by PARP inhibition to proton beam irradiation in cancer cells. Biochem. Biophys. Res. Commun..

[110] Calabrese C.R., Almassy R., Barton S., Batey M.A., Calvert A.H., Canan-Koch S., Durkacz B.W., Hostomsky Z., Kumpf R.A., Kyle S. (2004). Anticancer chemosensitization and radiosensitization by the novel poly(ADP-ribose) polymerase-1 inhibitor AG14361. J. Natl. Cancer Inst..

[111] Shelton J.W., Waxweiler T.V., Landry J., Gao H., Xu Y., Wang L., El-Rayes B., Shu H.K. (2013). In vitro and in vivo enhancement of chemoradiation using the oral PARP inhibitor ABT-888 in colorectal cancer cells. Int. J. Radiat. Oncol. Biol. Phys..

[112] Donawho C.K., Luo Y., Luo Y., Penning T.D., Bauch J.L., Bouska J.J., Bontcheva-Diaz V.D., Cox B.F., DeWeese T.L., Dillehay L.E. (2007). ABT-888, an orally active poly(ADP-ribose) polymerase inhibitor that potentiates DNA-damaging agents in preclinical tumor models. Clin. Cancer Res. Off. J. Am. Assoc. Cancer Res..

[113] Miura K., Sakata K., Someya M., Matsumoto Y., Matsumoto H., Takahashi A., Hareyama M. (2012). The combination of olaparib and camptothecin for effective radiosensitization. Radiat. Oncol..

[114] Waqar S.N., Robinson C., Olszanski A.J., Spira A., Hackmaster M., Lucas L., Sponton L., Jin H., Hering U., Cronier D. (2022). Phase I trial of ATM inhibitor M3541 in combination with palliative radiotherapy in patients with solid tumors. Investig. New Drugs.

[115] Czito B.G., Deming D.A., Jameson G.S., Mulcahy M.F., Vaghefi H., Dudley M.W., Holen K.D., DeLuca A., Mittapalli R.K., Munasinghe W. (2017). Safety and tolerability of veliparib combined with capecitabine plus radiotherapy in patients with locally advanced rectal cancer: A phase 1b study. Lancet Gastroenterol. Hepatol..

[116] Middleton M.R., Dean E., Evans T.R.J., Shapiro G.I., Pollard J., Hendriks B.S., Falk M., Diaz-Padilla I., Plummer R. (2021). Phase 1 study of the ATR inhibitor berzosertib (formerly M6620, VX-970) combined with gemcitabine ± cisplatin in patients with advanced solid tumours. Br. J. Cancer.

[117] Shapiro G.I., Wesolowski R., Devoe C., Lord S., Pollard J., Hendriks B.S., Falk M., Diaz-Padilla I., Plummer R., Yap T.A. (2021). Phase 1 study of the ATR inhibitor berzosertib in combination with cisplatin in patients with advanced solid tumours. Br. J. Cancer.

[118] Yap T.A., O’Carrigan B., Penney M.S., Lim J.S., Brown J.S., de Miguel Luken M.J., Tunariu N., Perez-Lopez R., Rodrigues D.N., Riisnaes R. (2020). Phase I Trial of First-in-Class ATR Inhibitor M6620 (VX-970) as Monotherapy or in Combination with Carboplatin in Patients With Advanced Solid Tumors. J. Clin. Oncol. Off. J. Am. Soc. Clin. Oncol..

[119] Bendell J.C., Bischoff H.G., Hwang J., Reinhardt H.C., Zander T., Wang X., Hynes S., Pitou C., Campbell R., Iversen P. (2020). A phase 1 dose-escalation study of checkpoint kinase 1 (CHK1) inhibitor prexasertib in combination with p38 mitogen-activated protein kinase (p38 MAPK) inhibitor ralimetinib in patients with advanced or metastatic cancer. Investig. New Drugs.

[120] Moore K.N., Hong D.S., Patel M.R., Pant S., Ulahannan S.V., Jones S., Meric-Bernstam F., Wang J.S., Aljumaily R., Hamilton E.P. (2021). A Phase 1b Trial of Prexasertib in Combination with Standard-of-Care Agents in Advanced or Metastatic Cancer. Target. Oncol..

[121] Hong D., Infante J., Janku F., Jones S., Nguyen L.M., Burris H., Naing A., Bauer T.M., Piha-Paul S., Johnson F.M. (2016). Phase I Study of LY2606368, a Checkpoint Kinase 1 Inhibitor, in Patients With Advanced Cancer. J. Clin. Oncol. Off. J. Am. Soc. Clin. Oncol..

[122] Plummer E.R., Kristeleit R.S., Cojocaru E., Haris N.M., Carter L., Jones R.H., Blagden S.P., Evans T.R.J., Arkenau H.-T., Sarker D. (2019). A first-in-human phase I/II trial of SRA737 (a Chk1 Inhibitor) in subjects with advanced cancer. J. Clin. Oncol..

[123] Italiano A., Infante J.R., Shapiro G.I., Moore K.N., LoRusso P.M., Hamilton E., Cousin S., Toulmonde M., Postel-Vinay S., Tolaney S. (2018). Phase I study of the checkpoint kinase 1 inhibitor GDC-0575 in combination with gemcitabine in patients with refractory solid tumors. Ann. Oncol. Off. J. Eur. Soc. Med. Oncol..

[124] Sausville E., Lorusso P., Carducci M., Carter J., Quinn M.F., Malburg L., Azad N., Cosgrove D., Knight R., Barker P. (2014). Phase I dose-escalation study of AZD7762, a checkpoint kinase inhibitor, in combination with gemcitabine in US patients with advanced solid tumors. Cancer Chemother. Pharmacol..

[125] Seto T., Esaki T., Hirai F., Arita S., Nosaki K., Makiyama A., Kometani T., Fujimoto C., Hamatake M., Takeoka H. (2013). Phase I, dose-escalation study of AZD7762 alone and in combination with gemcitabine in Japanese patients with advanced solid tumours. Cancer Chemother. Pharmacol..

[126] Ho A.L., Bendell J.C., Cleary J.M., Schwartz G.K., Burris H.A., Oakes P., Agbo F., Barker P.N., Senderowicz A.M., Shapiro G. (2011). Phase I, open-label, dose-escalation study of AZD7762 in combination with irinotecan (irino) in patients (pts) with advanced solid tumors. J. Clin. Oncol..

[127] Leijen S., van Geel R.M., Pavlick A.C., Tibes R., Rosen L., Razak A.R., Lam R., Demuth T., Rose S., Lee M.A. (2016). Phase I Study Evaluating WEE1 Inhibitor AZD1775 As Monotherapy and in Combination With Gemcitabine, Cisplatin, or Carboplatin in Patients With Advanced Solid Tumors. J. Clin. Oncol. Off. J. Am. Soc. Clin. Oncol..

[128] Do K., Wilsker D., Ji J., Zlott J., Freshwater T., Kinders R.J., Collins J., Chen A.P., Doroshow J.H., Kummar S. (2015). Phase I Study of Single-Agent AZD1775 (MK-1775), a Wee1 Kinase Inhibitor, in Patients With Refractory Solid Tumors. J. Clin. Oncol. Off. J. Am. Soc. Clin. Oncol..

[129] Cohen D.J., Grabocka E., Bar-Sagi D., Godin R., Leichman L.P. (2017). A phase Ib study combining irinotecan with AZD1775, a selective WEE 1 kinase inhibitor, in RAS/RAF mutated metastatic colorectal cancer patients who progressed on first line therapy. J. Clin. Oncol..

[130] Berlin J., Ramanathan R.K., Strickler J.H., Subramaniam D.S., Marshall J., Kang Y.K., Hetman R., Dudley M.W., Zeng J., Nickner C. (2018). A phase 1 dose-escalation study of veliparib with bimonthly FOLFIRI in patients with advanced solid tumours. Br. J. Cancer.

[131] Chen E.X., Jonker D.J., Siu L.L., McKeever K., Keller D., Wells J., Hagerman L., Seymour L. (2016). A Phase I study of olaparib and irinotecan in patients with colorectal cancer: Canadian Cancer Trials Group IND 187. Investig. New Drugs.

[132] Gorbunova V., Beck J.T., Hofheinz R.D., Garcia-Alfonso P., Nechaeva M., Cubillo Gracian A., Mangel L., Elez Fernandez E., Deming D.A., Ramanathan R.K. (2019). A phase 2 randomised study of veliparib plus FOLFIRI±bevacizumab versus placebo plus FOLFIRI±bevacizumab in metastatic colorectal cancer. Br. J. Cancer.

[133] Kummar S., Chen A., Ji J., Zhang Y., Reid J.M., Ames M., Jia L., Weil M., Speranza G., Murgo A.J. (2011). Phase I study of PARP inhibitor ABT-888 in combination with topotecan in adults with refractory solid tumors and lymphomas. Cancer Res..

[134] Leichman L., Groshen S., O’Neil B.H., Messersmith W., Berlin J., Chan E., Leichman C.G., Cohen S.J., Cohen D., Lenz H.J. (2016). Phase II Study of Olaparib (AZD-2281) After Standard Systemic Therapies for Disseminated Colorectal Cancer. Oncol..

[135] Pishvaian M.J., Slack R.S., Jiang W., He A.R., Hwang J.J., Hankin A., Dorsch-Vogel K., Kukadiya D., Weiner L.M., Marshall J.L. (2018). A phase 2 study of the PARP inhibitor veliparib plus temozolomide in patients with heavily pretreated metastatic colorectal cancer. Cancer.

[136] Samol J., Ranson M., Scott E., Macpherson E., Carmichael J., Thomas A., Cassidy J. (2012). Safety and tolerability of the poly(ADP-ribose) polymerase (PARP) inhibitor, olaparib (AZD2281) in combination with topotecan for the treatment of patients with advanced solid tumors: A phase I study. Investig. New Drugs.

[137] Smith G., Alholm Z., Coleman R.L., Monk B.J. (2021). DNA Damage Repair Inhibitors-Combination Therapies. Cancer J..

[138] Fuchss T., Grädler U., Schiemann K., Kuhn D., Kubas H., Dahmen H., Zimmermann A., Zenke F., Blaukat A. (2019). Abstract 3500: Highly potent and selective ATM kinase inhibitor M4076: A clinical candidate drug with strong anti-tumor activity in combination therapies. Cancer Res..

[139] Seligmann J.F., Fisher D.J., Brown L.C., Adams R.A., Graham J., Quirke P., Richman S.D., Butler R., Domingo E., Blake A. (2021). Inhibition of WEE1 Is Effective in TP53- and RAS-Mutant Metastatic Colorectal Cancer: A Randomized Trial (FOCUS4-C) Comparing Adavosertib (AZD1775) With Active Monitoring. J. Clin. Oncol. Off. J. Am. Soc. Clin. Oncol..

[140] Cuneo K.C., Morgan M.A., Sahai V., Schipper M.J., Parsels L.A., Parsels J.D., Devasia T., Al-Hawaray M., Cho C.S., Nathan H. (2019). Dose Escalation Trial of the Wee1 Inhibitor Adavosertib (AZD1775) in Combination With Gemcitabine and Radiation for Patients With Locally Advanced Pancreatic Cancer. J. Clin. Oncol. Off. J. Am. Soc. Clin. Oncol..

[141] Van Bijsterveldt L., Durley S.C., Maughan T.S., Humphrey T.C. (2021). The Challenge of Combining Chemo- and Radiotherapy with Checkpoint Kinase Inhibitors. Clin. Cancer Res. Off. J. Am. Assoc. Cancer Res..

[142] Mansoori B., Mohammadi A., Davudian S., Shirjang S., Baradaran B. (2017). The Different Mechanisms of Cancer Drug Resistance: A Brief Review. Adv. Pharm. Bull..

[143] Fok J.H.L., Ramos-Montoya A., Vazquez-Chantada M., Wijnhoven P.W.G., Follia V., James N., Farrington P.M., Karmokar A., Willis S.E., Cairns J. (2019). AZD7648 is a potent and selective DNA-PK inhibitor that enhances radiation, chemotherapy and olaparib activity. Nat. Commun..

[144] Overman M.J., Lonardi S., Wong K.Y.M., Lenz H.J., Gelsomino F., Aglietta M., Morse M.A., Van Cutsem E., McDermott R., Hill A. (2018). Durable Clinical Benefit With Nivolumab Plus Ipilimumab in DNA Mismatch Repair-Deficient/Microsatellite Instability-High Metastatic Colorectal Cancer. J. Clin. Oncol. J. Am. Soc. Clin. Oncol..

[145] De’ Angelis G.L., Bottarelli L., Azzoni C., De’ Angelis N., Leandro G., Di Mario F., Gaiani F., Negri F. (2018). Microsatellite instability in colorectal cancer. Acta Bio-Med. Atenei Parm..

[146] Demaria S., Formenti S.C. (2012). Radiation as an immunological adjuvant: Current evidence on dose and fractionation. Front. Oncol..

[147] Herskind C., Wenz F., Giordano F.A. (2017). Immunotherapy Combined with Large Fractions of Radiotherapy: Stereotactic Radiosurgery for Brain Metastases-Implications for Intraoperative Radiotherapy after Resection. Front. Oncol..

[148] Apetoh L., Ghiringhelli F., Tesniere A., Obeid M., Ortiz C., Criollo A., Mignot G., Maiuri M.C., Ullrich E., Saulnier P. (2007). Toll-like receptor 4-dependent contribution of the immune system to anticancer chemotherapy and radiotherapy. Nat. Med..

[149] Ahmed A., Tait S.W.G. (2020). Targeting immunogenic cell death in cancer. Mol. Oncol..

[150] Theelen W., Chen D., Verma V., Hobbs B.P., Peulen H.M.U., Aerts J., Bahce I., Niemeijer A.L.N., Chang J.Y., de Groot P.M. (2021). Pembrolizumab with or without radiotherapy for metastatic non-small-cell lung cancer: A pooled analysis of two randomised trials. Lancet Respir. Med..

[151] Golden E.B., Apetoh L. (2015). Radiotherapy and immunogenic cell death. Semin. Radiat. Oncol..

[152] Mouw K.W., Goldberg M.S., Konstantinopoulos P.A., D’Andrea A.D. (2017). DNA Damage and Repair Biomarkers of Immunotherapy Response. Cancer Discov..

[153] Ragu S., Matos-Rodrigues G., Lopez B.S. (2020). Replication Stress, DNA Damage, Inflammatory Cytokines and Innate Immune Response. Genes.

[154] Song Y., Huang J., Liang D., Hu Y., Mao B., Li Q., Sun H., Yang Y., Zhang J., Zhang H. (2020). DNA Damage Repair Gene Mutations Are Indicative of a Favorable Prognosis in Colorectal Cancer Treated With Immune Checkpoint Inhibitors. Front. Oncol..

[155] Storozynsky Q., Hitt M.M. (2020). The Impact of Radiation-Induced DNA Damage on cGAS-STING-Mediated Immune Responses to Cancer. Int. J. Mol. Sci..

[156] Zheng J., Mo J., Zhu T., Zhuo W., Yi Y., Hu S., Yin J., Zhang W., Zhou H., Liu Z. (2020). Comprehensive elaboration of the cGAS-STING signaling axis in cancer development and immunotherapy. Mol. Cancer.

[157] Carter L. (2016). Phase I modular study of AZD6738, a novel oral, potent and selective ataxia telangiectasia Rad3-related (ATR) inhibitor in combination (combo) with carboplatin, olaparib or durvalumab in patients (pts) with advanced cancers. Eur. J. Cancer.

[158] Ahmad S.S., Crittenden M.R., Tran P.T., Kluetz P.G., Blumenthal G.M., Bulbeck H., Baird R.D., Williams K.J., Illidge T., Hahn S.M. (2019). Clinical Development of Novel Drug-Radiotherapy Combinations. Clin. Cancer Res. Off. J. Am. Assoc. Cancer Res..

[159] Vendetti F.P., Karukonda P., Clump D.A., Teo T., Lalonde R., Nugent K., Ballew M., Kiesel B.F., Beumer J.H., Sarkar S.N. (2018). ATR kinase inhibitor AZD6738 potentiates CD8+ T cell-dependent antitumor activity following radiation. J. Clin. Investig..

