# Supplementary material for: Targeting the DNA Damage Response and DNA Repair Pathways to Enhance Radiosensitivity in Colorectal Cancer
Source: Cancers (Basel). 2022 Oct 5;14(19):4874. doi: 10.3390/cancers14194874 (PMC9561988; doi:10.3390/cancers14194874)
Supplement: Supplementary file 1 [file cancers-14-04874-s001.zip › cancers-1841675-supplementary.pdf]

Table S1: Targeted proteins involved in the DDR and DNA repair pathways in response to IR.

**Protein Name**	**Length (amino acids)**	**Activity Regulation**	**Primary Biological Process**
ATM	3056	Activated with DNA in the presence of MRN	DNA damage checkpoint, cell cycle arrest, cell response to IR, DSBs repair
ATR	2644	Directed to RPA, stimulated by TopBP1	DNA damage checkpoint, cell response to IR, signal transduction, DNA repair, regulator of DNA replication stress and genome integrity
CHK1	476	Activated through phosphorylation	DNA damage checkpoint, cell cycle arrest, cell response to IR, DSBs repair by HR and NHEJ, DNA replication
CHK2	543	Activated through phosphorylation	DNA damage checkpoint, cell cycle arrest, DNA repair and cell apoptosis in response to DSBs
WEE1	646	Activated through phosphorylation	G2 to M phase transition, negative regulation of G1 to S phase transition, establishment of cell polarity, cell division
DNA-PKcs	4128	Autophosphorylation or phosphorylated via ATM	Negative regulation of response to IR, DSBs repair
PARP1	1014	NAD+ as a substrate	Response to IR, detection of SSBs, DNA repair and stabilization of replication forks

DDR, DNA damage response; IR, ionizing radiation; DSBs, double-strand breaks; MRN, Mre11–Rad50–NBS1; HR, homologous recombination; NHEJ, nonhomologous end joining; RPA, replication protein A; TopBP1, DNA topoisomerase 2-binding protein 1.
